# Sexual Size Dimorphism Correlates With the Number of Androgen Response Elements in Mammals, But Only in Small-Bodied Species

**DOI:** 10.1093/gbe/evaf068

**Published:** 2025-04-18

**Authors:** Caleb R Ghione, Matthew D Dean

**Affiliations:** Molecular and Computational Biology, University of Southern California, Los Angeles, CA, USA; Molecular and Computational Biology, University of Southern California, Los Angeles, CA, USA

**Keywords:** sexual size dimorphism, hormone response elements, androgen signaling, genetics, evolution

## Abstract

Sexual size dimorphism is common throughout the animal kingdom, but its evolution and development remain difficult to explain given most of the genome is shared between males and females. Sex-biased regulation of genes via sex hormone signaling offers an intuitive mechanism by which males and females could develop different body sizes. One prediction of this hypothesis is that the magnitude of sexual size dimorphism scales with the number of androgen response elements or estrogen response elements, the DNA motifs to which sex hormone receptors bind. Here, we test this hypothesis using 268 mammalian species with full genome assemblies and annotations. We find that in the two smallest-bodied lineages (Chiroptera and Rodentia), sexual size dimorphism increases (male-larger) as the number of androgen response elements in a genome increases. In fact, myomorph rodents—which are especially small-bodied with high sexual size dimorphism—show an explosion of androgen receptor elements in their genomes. In contrast, the three large-bodied lineages (orders Carnivora, Cetartiodactyla, and Primates) do not show this relationship, instead following Rensch's Rule, or the observation that sexual size dimorphism increases with overall body size. One hypothesis to unify these observations is that small-bodied organisms like bats and rodents tend to reach peak reproductive fitness quickly and are more reliant on hormonal signaling to achieve sexual size dimorphism over relatively short time periods. Our study uncovers a previously unappreciated relationship between sexual size dimorphism, body size, and hormone signaling that likely varies in ways related to life history.

**Significance**

How does sexual size dimorphism, where males and females develop different body sizes, evolve? Since males and females share most of their genome, sexual size dimorphism must arise from sex-specific regulation of the genome, for example via changes in the number of sites in the genome that bind sex hormone receptors. Across 268 mammal species with complete genomes, we find that species with the highest sexual size dimorphism (male-larger) tend to harbor more androgen binding elements near genes, but only in small-bodied lineages. Small body size is often correlated with short lifespans and quick reproductive development; our study suggests that small species rely on hormonal signaling to achieve sexual size dimorphism quickly. Because large-bodied species can accumulate body size over multiple seasons, they may be less reliant on hormonal signaling to achieve sexual size dimorphism. Our study sheds light on the evolution and development of sexual size dimorphism.

## Introduction

Sexual size dimorphism (SSD) refers to the degree by which males and females of the same species differ in body size and is a common feature across many groups including mammals (Ralls 1977; Fairbairn et al. 2007; Lindenfors et al. 2007). Male-larger species might indicate selection favors relatively large males in contests with other males (Clutton-Brock et al. 1977; Lindenfors and Tullberg 1998; Weckerly 1998; Lindenfors et al. 2002; Perez-Barberia et al. 2002), while female-larger species could arise if relatively large females have increased fecundity (Darwin 1872; Ralls 1976). SSD could also arise if selection favors niche differentiation to reduce competition between the sexes (Cox and Calsbeek 2009).

The very existence of SSD implies sexual conflict over body size, and understanding its evolution is critical for deciphering resolutions to this conflict. One potential resolution could be hormonal regulation. Even though most of the genome is shared between males and females, sex hormones might enable sex-biased regulation of genes involved in growth. Androgen and estrogen circulate at different levels in males and females, and their main downstream targets are a single *Androgen receptor* (*Ar*) and two different *Estrogen receptors* (*Erα* and *Erβ*). Upon binding to their hormonal ligands, these receptors undergo conformational changes, bind to particular DNA motifs, and directly or indirectly modify the expression of nearby genes (Claessens et al. 2001; Sanchez et al. 2002; Verrijdt et al. 2003; McEwan 2004; Robins 2005; Bourdeau et al. 2008). Thus, androgen and estrogen receptors typically act as transcription factors that regulate many genes in a sex-biased manner (Ellegren and Parsch 2007; Hau 2007; Parsch and Ellegren 2013; Ingleby et al. 2014; Kitano et al. 2020), offering a potential molecular mechanism to achieve sex-biased size (Ellegren and Parsch 2007; Hahn 2007; Smith et al. 2013; Ingleby et al. 2014; Mayne et al. 2016; Naqvi et al. 2019; Hale et al. 2022).

If hormonal regulation enables SSD, the magnitude of SSD should correlate with the number of DNA motifs that act as androgen response elements (AREs) or estrogen response elements (EREs). Here, we test this hypothesis by analyzing body size and genome content across 268 mammalian species from the Zoonomia Project (Zoonomia 2020; Christmas et al. 2023; Foley et al. 2023). We uncover a distinct difference in this relationship across five orders of mammals. In the relatively small-bodied orders Chiroptera and Rodentia, SSD increased (male-larger) with the number of AREs in their genomes. Interestingly, the subclade of 21 myomorph rodent species was especially small-bodied but with high SSD, and this clade showed an explosion of AREs. SSD in relatively larger-bodied species from three orders (Carnivora, Cetartiodactyla, and Primates) did not correlate to ARE or ERE counts, and instead followed Rensch's Rule, which refers to the observation that SSD in male-larger species tends to increase with increasing body size while the magnitude of SSD in female-larger species tends to decrease.

Our study suggests that different species deploy different developmental mechanisms to achieve sex-biased optima, which in turn might help resolve sexual conflict over body size. Because small-bodied species tend to have short lifespans and reach reproductive maturity quickly, they may be more reliant on hormonal signaling to achieve SSD in relatively short periods of time. In contrast, large-bodied species may accumulate SSD over multiple seasons and thus are less reliant on hormonal signaling. Our study reveals how SSD varies across the phylogeny and how sex-specific regulation may be related to differences in life history.

## Results

Of 455 unique TOGA-annotated species from Zoonomia, 268 overlapped a mammalian phylogeny (Upham et al. 2019) and had reliable body size data in the literature (supplementary Files S1 and S2, Supplementary Material online). Among these 268 species, SSD ranged from −0.99 (dugongs) to 1.55 (southern elephant seals). Positive SSD values indicate male-larger species, while negative SSD values indicate female-larger species. Within 50 kb upstream/downstream of a transcription start site (TSS), we counted between 501 and 40,891 AREs (mean = 4,692) and between 12 and 957 (mean = 162.5) EREs across species. The number of hormone response elements (HREs) correlated with scaffold N50 (phlylom *P*-value < 10^−4^), an indirect proxy for genome quality. However, lower genome quality correlated with more HREs, indicating that our results were not sensitive to differences in genome quality.

**Fig. 1.**
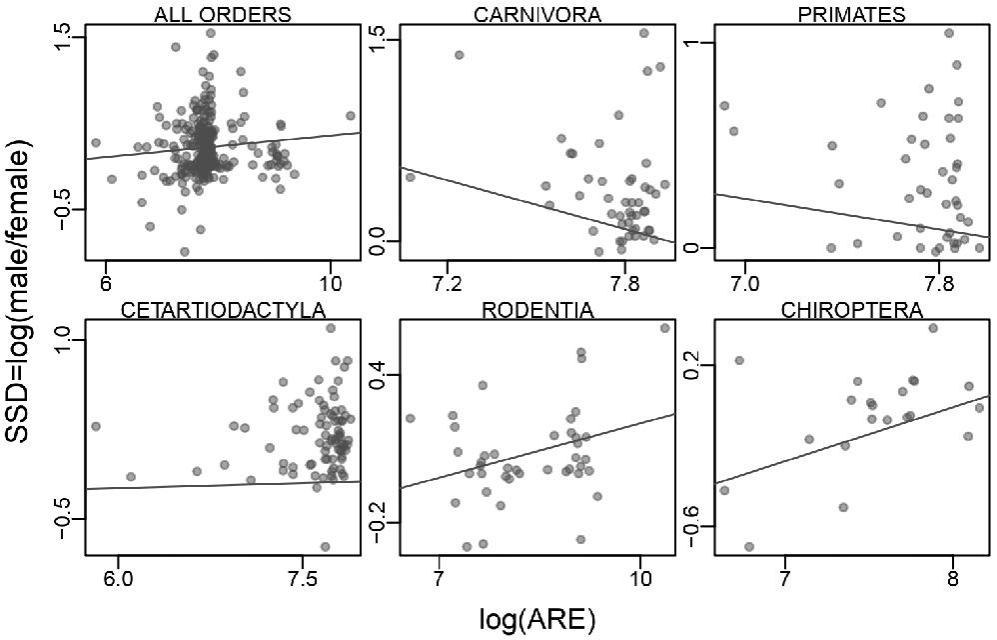
The correlations between SSD and total ARE counts among orders. ARE counts 50 kb upstream/downstream of a TSS, natural-log-transformed. Each point on the plot is a species, and the slope of the line is derived from phylogenetically controlled linear models (see methods).

**Fig. 2.**
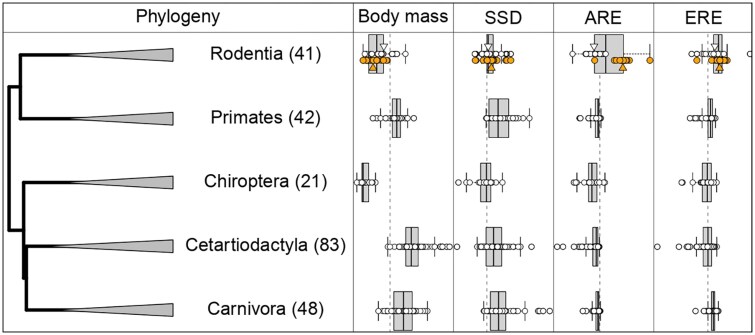
The five orders represented by at least 20 species (number in parentheses). Relationship between average body mass, SSD, ARE counts, and ERE counts. For SSD, the vertical dashed line indicates SSD = 0, species to the right are male-larger species to the left are female-larger. For all other phenotypes, the vertical dashed lines indicate phylogenetic mean estimated at the root of the mammalian tree. Rodentia has been divided into two groups for visualization: the second row of orange dots are myomorph rodents, showing their dramatic increase in AREs near genes. Small triangles indicate the respective Rodentia group's means. ARE and ERE counts 50 kb upstream/downstream of a TSS, natural-log-transformed. The most recent common ancestor in this tree occurred 72 million years ago. The most recent common ancestor for each order occurred between 49 and 57 million years ago.

We employed phylogenetically controlled linear models to test whether the magnitude of SSD covaried with the number of AREs/EREs and overall body size (SSD ∼ log(ARE-or-ERE) + log(body_size)). When all species were included, we detected a significantly positive relationship between SSD and body size (*P* = 0.001) but not the number of AREs (*P* = 0.179) or EREs (*P* = 0.310) (Table 1). Closer inspection showed that these relationships varied across mammalian orders (Table 1; Figs. 1 and 2), so we re-analyzed the five orders that were represented by at least 20 species. We could not simply include “order” as a covariate in the linear model because the scale over which SSD varies within each order is too different among groups. These five orders share almost no evolutionary history and represent hundreds of millions of years of independent evolution (most recent common ancestor of five orders in our dataset range 49 to 57 million years ago), justifying their separate analyses (Fig. 2).

**Table 1:** Phylolm results [SSD ∼ log(ARE or ERE) + log(body_size)] for genomic regions within 50 kb of TSS

Order	*N*	HRE	mean (Sd)	HRE effect	HRE *P-*value	Body size effect	Body size *P-*value	Lambda	R.squared
	species	type	log HRE						
All	268	ARE	8.01 (0.49)	0.066	0.179	0.030	0.001	0.694	0.049
Carnivora	48		7.95 (0.16)	−0.476	0.110	0.067	0.017	0.353	0.153
Cetartiodactyla	83		7.80 (0.32)	0.039	0.668	0.062	0.005	0.811	0.097
Chiroptera	21		7.74 (0.39)	0.305	0.034	−0.019	0.747	0.104	0.228
Primates	42		7.94 (0.21)	−0.221	0.196	0.064	0.049	0.437	0.137
Rodentia	41		8.54 (0.79)	0.078	0.050	0.006	0.687	0.000	0.099
All	268	ERE	4.72 (0.39)	0.054	0.310	0.029	0.001	0.695	0.046
Carnivora	48		4.84 (0.16)	−0.336	0.265	0.064	0.043	0.643	0.099
Cetartiodactyla	83		4.54 (0.38)	0.037	0.663	0.061	0.005	0.809	0.096
Chiroptera	21		4.52 (0.38)	0.394	0.004	−0.038	0.521	1.000	0.388
Primates	42		4.71 (0.24)	−0.156	0.299	0.066	0.050	0.494	0.121
Rodentia	41		5.09 (0.41)	0.015	0.839	−0.004	0.808	0.000	0.003

Lambda = Pagel's lambda, an estimate of phylogenetic inertia. R.squared = proportion of variance in SSD explained by the model, phylogenetically controlled.

### In Relatively Large-Bodied Species, SSD Follows Rensch's Rule But Is Not Correlated to AREs or EREs

Carnivora, Cetartiodactyla, and Primates all followed Rensch's Rule, where the magnitude of SSD increased with increasing body size (*P* < 0.017, 0.005, and 0.049, respectively) (Table 1). None of these groups showed a relationship between SSD and the number of AREs or EREs within 50 kb of a TSS. Anderson and Jones (2024) also found no relationship between SSD and AREs in a study of 26 primate species. These results remain largely unchanged with genomic windows of 10, 100, or 1,000 kb (supplementary File S3, Supplementary Material online).

### In Relatively Small-Bodied Species, SSD Is Positively Correlated With AREs But Does Not Follow Rensch's Rule

In the two smallest-bodied lineages, Chiroptera and Rodentia, SSD was positively correlated with the number of AREs 50 kb upstream/downstream of a TSS (*P* = 0.034 and 0.050, respectively, Table 1), but did not follow Rensch's Rule (*P* = 0.747 and 0.687, respectively, Table 1). These results remain largely unchanged if we change the genomic window from within 50 kb upstream/downstream of a TSS to 10, 100, or 1,000 kb (supplementary File S3, Supplementary Material online).

The proportion of variance in SSD explained in each linear model averaged 12.6% (range 0.3 to 22.8, Table 1). This might be interpreted as a small amount of variance explained. However, the nature of our investigation—where a single body size for males and females is assigned per species and we assume that function is related to HRE motif counts alone—is likely to introduce noise. Therefore, our intuition is that the true relationship between SSD and HRE counts could be much stronger than we detect here.

Interestingly, the positive correlation between SSD and AREs in Rodentia was accompanied by a dramatic increase in the number of AREs in their genomes compared with the other four orders (Fig. 2). Further exploration showed that this pattern was largely driven by the monophyletic clade of myomorph rodents (orange points in Fig. 2). Specifically, the 21 myomorph species had nearly four times as many AREs, on average, compared with the 20 non-myomorph rodent species (13,188 vs. 3,400) (Fig. 2). Myomorph rodents also had an average SSD nearly three times that of non-myomorph rodents (SSD = 0.11 vs. 0.03) (Fig. 2), while at the same time being an order of magnitude smaller (0.23 vs. 3.83 kg) (Fig. 2).

Padilla-Morales et al. (2024) observed an increase in the number of some gene families as a function of SSD across mammals, especially in genes involved in pheromone detection. Therefore, we tested whether the explosion of AREs in myomorph rodents simply reflects an increase in the total size of the genome that was interrogated across species. This was not a likely explanation: within 50 kb upstream/downstream of a TSS, 941 Mb was interrogated from myomorph rodents on average, while 776 Mb was interrogated from non-myomorph rodents.

### Principal Components Analysis

Transforming counts of the 18 different ARE motifs into principal components did not add any additional insight. PC1 of log-transformed ARE and ERE counts was generally uncorrelated with SSD. Under all models, there were only four significant relationships: Carnivora at 100 kb and Chiroptera at 50, 100, and 1,000 kb (supplementary File S3, Supplementary Material online).

### Gene-Centric Analyses

**Fig. 3.**
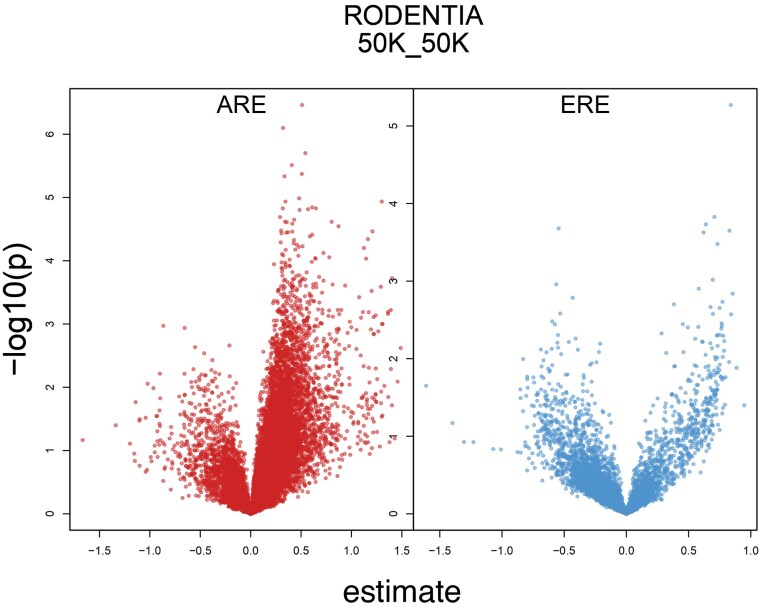
Gene-centric analyses of the linear model SSD ∼ log(HRE) + log(body_size). Each point is a gene represented by at least ten rodent species that show variability in HRE counts within 50 kb of TSS. The *x*-axis indicates the effect of HRE count on SSD. The *y*-axis indicates the −log_10_ × *P*-value. For AREs, a large majority of genes, regardless of statistical significance, have a positive influence on SSD. This pattern contrasts with EREs, where the majority of genes have a negative impact on SSD.

Next, we tested each gene separately for a correlation between its AREs/EREs and SSD, again using average body size as a covariate. The order Rodentia consistently showed an excess of genes that had a positive effect on SSD. For AREs within 50 kb of a TSS, 18,299 genes could be tested, meaning that the gene was represented by at least 10 rodent species that show variability in ARE counts. Of these, 14,363 (78%) showed a positive relationship between their number of AREs and SSD (Fig. 3). This pattern held for the 10, 100, and 1,000 kb windows (69.0%, 82%, and 88% of genes with a positive effect on SSD, respectively). Interestingly, the opposite pattern was observed for EREs (Fig. 3). For the order Rodentia, 73%, 66%, 63%, and 47% of genes (for the 10, 50, 100, and 1,000 kb cutoffs, respectively) had a *negative* effect on SSD. The pattern was less consistent among the other mammalian orders (supplementary Files S4 to S6, Supplementary Material online).

Given this result, we used Gene Ontology analyses to test whether genes with a significantly positive effect between their AREs and SSD (uncorrected *P* < 0.05) were enriched for annotated functions. Under the Biological Process ontology, we detected an enrichment of genes with functions “RNA metabolic process” and “cellular metabolic process” (supplementary File S7, Supplementary Material online). We did not detect any enrichment of genes involved in olfactory processes, as might be predicted by the recent finding that species with higher SSD showed an expansion of such gene families (Padilla-Morales et al. 2024).

## Discussion

Why do males and females within the same species often look different from each other? Many hypotheses have been proposed to explain why selection might favor different sizes in males and females (Cassini 2023), but it remains poorly understood *how* males and females develop different body sizes since most protein coding genes occur in both sexes. Here, we combined modern genomic data with traditional phylogenetic methodology to re-examine SSD in the context of hormonal signaling. In small-bodied lineages, SSD correlated with AREs but not overall body size, while large-bodied lineages followed Rensch's Rule without an association with AREs or EREs.

Body size is correlated with numerous physiological and life-history features that might give insight into the patterns we observe (Stearns 1998). Small-bodied species tend to reach sexual maturity quickly, living relatively short lives under relatively high metabolic rates (Schmidt-Nielsen 1984; Peters 1986; Promislow 1991; Gaillard et al. 1994; Gillooly et al. 2001; Gillooly et al. 2002; Speakman 2005; Bonduriansky et al. 2008; Curlis et al. 2021; Kuparinen et al. 2023), although there are many exceptions (Jurgens and Prothero 1987; Podlutsky et al. 2005; Seim et al. 2013; Diaz 2020). All else equal, we expect large-bodied species should therefore have more absolute time to amass differences in body size. In some cases, the larger sex takes longer to reach sexual maturity than the smaller sex (Wiley 1974; Shea 1985; Kozlowski 1989; Leigh 1992; Badyaev 2002; Teder and Blanckenhorn 2014), a strategy that is probably only available to long-lived species. Many rodent species live far less than 1 year—even if some of them can live for multiple years in a laboratory setting (Gorbunova et al. 2008).

While it is not yet possible to definitively assign differences in body size to genome-wide molecular mechanisms, our study suggests that hormonal signaling is an important mechanism for small-bodied species to achieve SSD relatively quickly. Since SSD increased with the number of AREs, one hypothesis is that males use AR binding to upregulate genes that directly or indirectly increase body size. Consistent with this hypothesis, the especially small, and presumably short-lived, myomorph rodents have experienced an explosion of AREs in their genomes compared with all other mammalian species including other rodents. Furthermore, most genes showed a positive effect of their number of AREs on SSD in rodents (Fig. 3), suggesting that most genes contribute to or are otherwise correlated with body size.

We currently lack the necessary data or methods to directly link hormonal signaling to SSD across these species, and the proxy we use (the number of AREs and EREs) suffers several limitations. First and foremost, the presence of a DNA motif does not necessarily mean it serves as a binding site for AR or ER. The 18 different AREs have variable binding specificity to AR and can be bound by transcription factors other than AR (De Umesono and Evans 1989; Nelson et al. 1999; Verrijdt et al. 2003; Shaffer et al. 2004; Denayer et al. 2010; Bruyn et al. 2011; Sahu et al. 2014). AREs sometimes act cooperatively to induce gene expression (Robins 2005), obscuring simple connections between the number of ARE motifs and the transcription of nearby genes. Chromatin structure will also impact the accessibility of hormone-binding elements to their receptors, so even if an ARE motif could impact gene expression, it might have little effect if buried in closed chromatin. Even if we knew which hormone receptors bound which elements, we would not know how gene expression is affected without further experimental manipulation, let alone how that variation contributes to variation in sex-biased body size. Perhaps such complexities explain why the relationship between androgen signaling and body size is sometimes paradoxical—for example, in two closely related species of lizards, testosterone had opposite effects on male body size (Cheverud et al. 1985; John-Alder et al. 2007). Nevertheless, Anderson and Jones (2024) showed that AREs congregated near male-biased genes and androgen-responsive genes, supporting the assumption that hormone-binding elements serve as a meaningful proxy for genomic regulation, at least with respect to AR. The uncertainty of using binding motifs as a proxy for genomic regulation should only add statistical noise to the relationship with SSD, and it is possible that the true correlations are much stronger than we are able to detect with our evolutionary analyses.

Because most of the genome is shared between males and females, genetic variation that influences body size is likely to evolve under sexual conflict, where an allele that increases body size benefits the larger sex to the detriment of the other. Our study explores mechanisms by which sexual conflict could be resolved, revealing different strategies that could be deployed across species. Our study suggests that small species rely more heavily on hormonal signaling to achieve SSD, while large species do not. Multiple studies have demonstrated sexually antagonistic genetic variation, including in mammals (Anunciado et al. 2001; Chan et al. 2012), flies (Pischedda and Chippindale 2006; Long and Rice 2007; Innocenti and Morrow 2010; Ruzicka et al. 2019), and others (Cox and Calsbeek 2009; Pennell and Morrow 2013), suggesting that conflict is never fully resolved. The existence of sexually antagonistic genetic variation likely poses constraints on the magnitude of SSD (Audet et al. 2024), leading Pennell and Morrow (2013) to coin the phrase “you can’t always get what you want”.

It is important to recognize that the mammalian lineages studied here differ not only in body size but also in the magnitude of their SSD. The three large-bodied lineages all have relatively large SSD (male-larger), ranging from an average of 0.195 (Cetartiodactyla) to 0.375 (Carnivora). In contrast, the small-bodied lineages have a fairly modest SSD of 0.073 (Rodentia) and in fact show the average female-larger SSD of −0.058 in Chiroptera, a pattern consistent with other studies (Lu et al. 2014; Tombak et al. 2024). Rensch's Rule applies to male-larger species, so it is perhaps unsurprising we do not observe it in these smaller-bodied lineages that have modest SSD. However, the positive correlation between SSD and AREs in both rodents and bats, the explosion of AREs in the especially small myomorph rodents, and the pervasive positive effect on SSD across genes, all suggest that small-bodied lineages are selected to achieve SSD, even if modest, through hormonal signaling.

Our study suggests that hormonal signaling is an important mechanism enabling males and females to approach different body size optima, but only in small-bodied species. Future studies linking the molecular biology of androgen signaling and body size promise to shed light on the fundamentally important evolution of body size in males versus females, and its potential relation to sexual conflict.

## Methods

All code is available in supplementary File S8, Supplementary Material online.

### Body Mass

We collected body mass data taken outside of breeding season, primarily to avoid data from pregnant or lactating females. Most body mass data were taken from Silva and Downing (1995), supplemented with additional literature sources (supplementary File S1, Supplementary Material online) (Silva and Downing 1995; Weckerly 1998; Bayart and Simmen 2005; Johnson et al. 2005; Thoren et al. 2006; Trillmich et al. 2006; Nazarova and Proskurnyak 2013; Brassey et al. 2018; Liker et al. 2021). Sexual size dimorphism (SSD) was calculated as *ln*(male/female) body mass, as recommended by Lovich and Gibbons (1992).

### Genome Quality

Some of our analyses could be confounded by variations in genome quality. For example, Kirilenko et al. (2023) annotated more genes from relatively high-quality genomes. We tested whether ARE or ERE counts correlated with contig N50, a common metric of genome quality, taken from Kirilenko et al. (2023). N50 is the contig length or longer which includes half the bases of the assembly.

### ARE and ERE Counts Across Whole Genomes

We counted AREs and EREs across all genome assemblies of the Zoonomia consortium, using genome annotations from the Zoonomia Project (Zoonomia 2020; Christmas et al. 2023; Foley et al. 2023). Zoonomia sequenced and assembled (or linked to genomes stored in NCBI) 524 genome assemblies from 464 unique species.

The canonical ARE is AGAACANNNTGTTCT, where the three N's indicate a three nucleotide spacer flanked by palindromic sequence (Roche et al. 1992; Pettersson et al. 1997; Gruber et al. 2004; Shaffer et al. 2004; De Bruyn et al. 2011). Seventeen additional AREs have been identified, all of which are similar in sequence but vary in their specificity of binding with AR. Following Anderson and Jones (2024), we analyzed all 18 AREs pooled. A single ERE has been identified as the sequence GGTCANNNTGACC (Roche et al. 1992; Claessens et al. 2001; Sanchez et al. 2002; Ikeda et al. 2015; Jones et al. 2019). We counted AREs and EREs using the Vcountpattern function from the Biostrings package in R (v 4.4.2) (Pages et al. 2024; R Core Team 2024). For any non-palindromic AREs, we added up the counts in the forward and reverse-complement directions of the genome.

### ARE and ERE Counts Near Protein Coding Regions

In addition to the whole genome counts, we focused on protein coding regions and their *cis*-regulatory regions. We used the gene annotations of Kirilenko et al. (2023), who developed *A Tool to infer Orthologs from Genome Alignments* (TOGA) (v. 1.1.8) (https://zoonomiaproject.org/the-data/) to essentially lift over annotations from a reference genome (the house mouse genome annotation *mm10*) to all other genome assemblies. TOGA makes six different gene calls: (i) *Intact* genes, where the middle 80% of the *mm10* reference gene is identified without inactivating mutations (frameshifts or early termination) in the target genome, (ii) *Partially intact* genes, which are intact genes that show gaps in the genome assembly, (iii) *Lost* genes, where the middle 80% of the *mm10* gene is present but contains at least one inactivating mutation, (iv) *Missing* genes, where less than 50% of the *mm10* gene is present, (v) *Uncertain lost* genes, which are genes that would be classified as *Lost* but where evidence is not strong, and (vi) *Paralogous projection* genes, which are *Intact* genes that are not orthologous to the reference *mm10* gene, for example a lineage-specific duplication of a gene.

We counted AREs and EREs that fell 10, 50, 100, or 1,000 kb upstream/downstream of TSSs of either *Intact* or *Paralogous Projection* genes. We included *Paralogous Projection* genes because our hypothesis was focused on the sex-biased expression of the genome, regardless of whether a gene was duplicated. We counted AREs and EREs once, even if they fell near multiple protein coding genes. The proximity over which AREs and EREs can affect gene expression is a topic of debate, but AREs have been shown to influence genes on the order of 1,000 kb away (Wilson et al. 2016). Reporter assays have also shown that a large number of AREs that regulate genes are within 50 kb of their respective TSS (Bolton et al. 2007). For this reason, we decided to use distances that could span the majority of AREs and EREs that are present in the genomes. We arrive at highly similar conclusions regardless of which cutoff we use, but present results based on the 50 kb cutoff in the main manuscript and discuss differences when they arise.

### Principal Component Analysis

We also explored the combined ARE and ERE counts in a principal component analysis. We first log_10_-transformed all ARE and ERE counts, then computed principal components after accounting for phylogenetic relationships, using the Phyl.pca function in the R package Phytools 2.0 (Revell 2012).

### Phylogenetically Controlled Linear Modeling

We implemented phylogenetically controlled linear models using the Phylolm function in the R package Phylolm version 2.6.5 (Ho and Ane 2014). The basic model tested was SSD∼ARE (or ERE) counts + body_size. SSD was calculated as *ln*(male/female body mass). ARE (or ERE) counts were counted from each genome. Body size was calculated as the average male + female body mass in each species. ARE (or ERE) counts and body size were *ln*-transformed prior to analyses. Average body size was included as a covariate because of “Rensch's Rule”, the observation that SSD in male-larger species increases with body size (Rensch 1994). In addition, many life-history traits covary with body size. We used the phylogeny of Upham et al. (2019), trimmed to match our data (supplementary File S2, Supplementary Material online).

### Gene-Centric Analyses

We also tested whether SSD correlated with the number of AREs or EREs on a gene-by-gene basis. This analysis represents an agnostic approach toward revealing genes or gene classes that explain variation in SSD. As above, we counted AREs and EREs that fell within 10, 50, 100, and 1,000 kb upstream or downstream of the TSS of *Intact* and *Paralogous Projection* genes. In this gene-centric approach only, AREs/EREs could be counted more than once if they fell near multiple genes. We only analyzed genes that were represented by at least 10 species and showed variability in ARE or ERE counts.

### Gene Ontology Analysis

Following our gene-centric analyses, we tested whether individual genes that were significantly positively correlated with SSD (uncorrected *P* < 0.05) were over-represented for Gene Ontology terms, using PantherDB version 19.0 (Mi et al. 2019).

## Supplementary Material

evaf068_Supplementary_Data

## Data Availability

All genomes used in this study are linked via NCBI in the Zoonomia consortium (https://zoonomiaproject.org/the-data/). All other data underlined in this article are available in the article and the uploaded supplementary files, Supplementary Material online. R scripts are available in supplementary File S8, Supplementary Material online.
