# Supplementary material for: Sexual Size Dimorphism Correlates With the Number of Androgen Response Elements in Mammals, But Only in Small-Bodied Species
Source: Genome Biol Evol. 2025 Apr 18;17(4):evaf068. doi: 10.1093/gbe/evaf068 (PMC12015095; doi:10.1093/gbe/evaf068)
Supplement: evaf068_Supplementary_Data [file evaf068_supplementary_data.zip › supplementary.3/supplementary/SuppFile4.reduced.pdf]

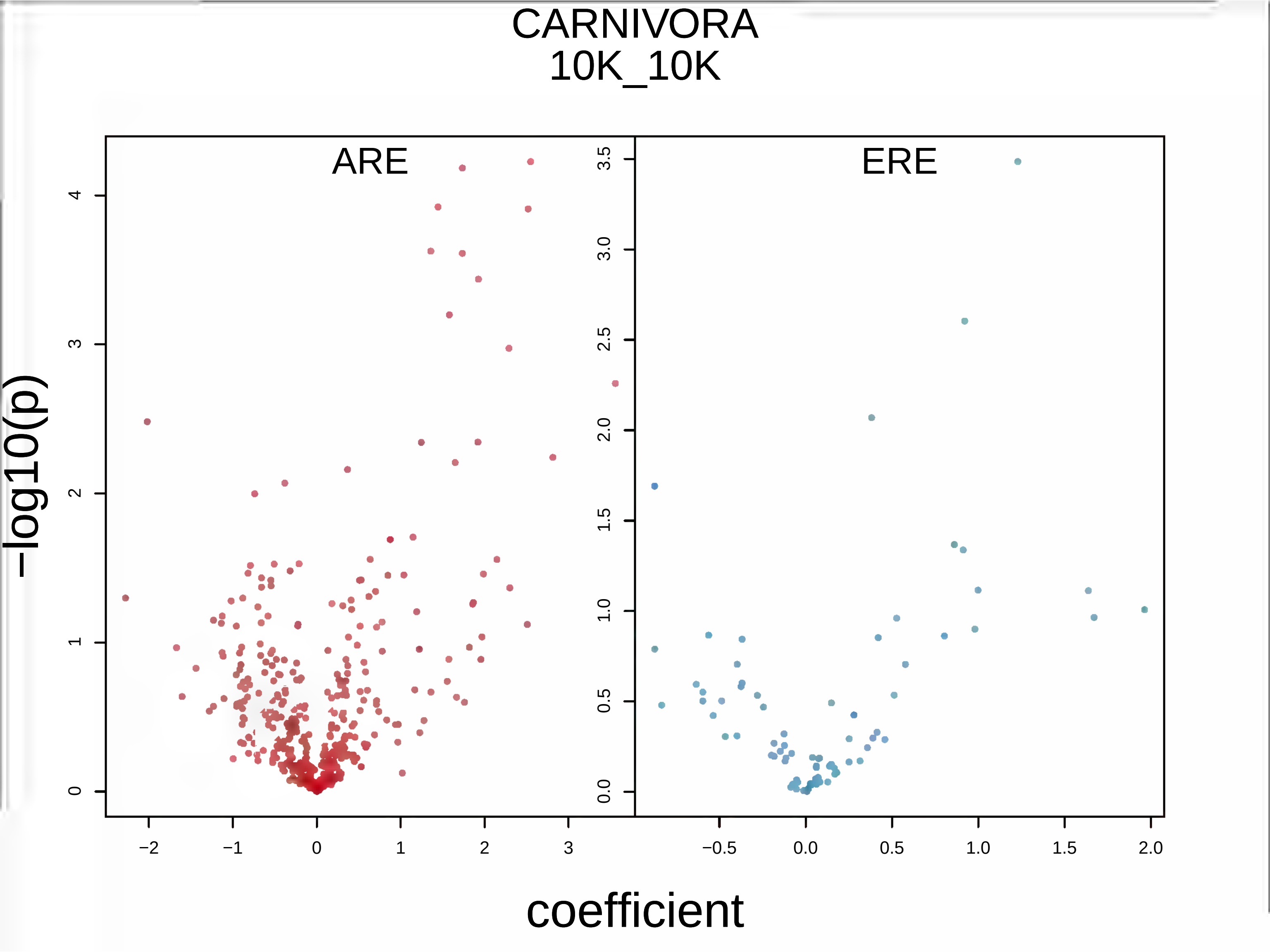
CARNIVORA
10K_10K
ARE
ERE
−log10(p)
coefficient
0
1
2
3
4
0.0
0.5
1.0
1.5
2.0
2.5
3.0
3.5
−2
−1
0
1
2
3
−0.5
0.0
0.5
1.0
1.5
2.0

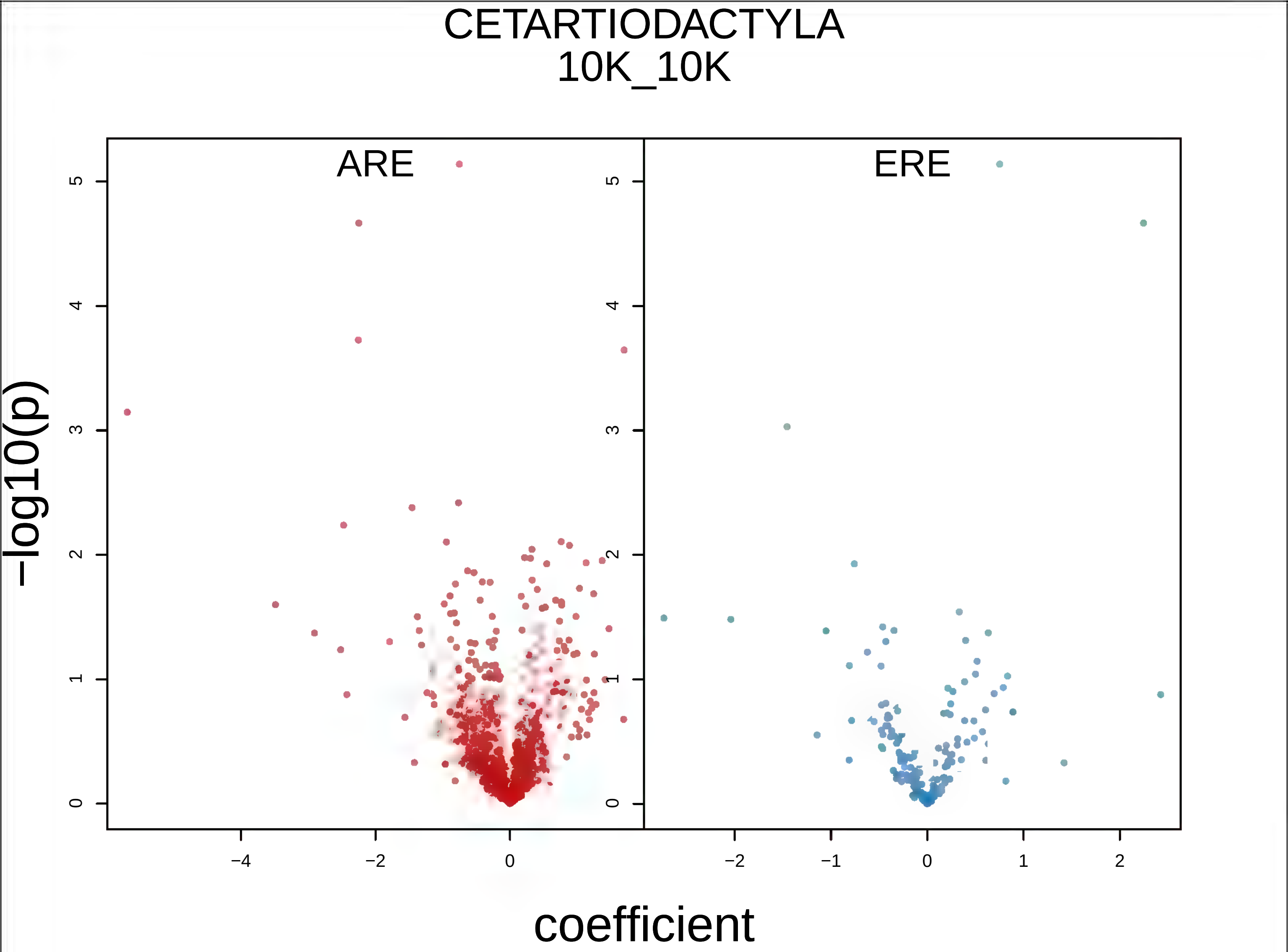

CETARTIODACTYLA
10K_10K
ARE
ERE
−log10(p)
coefficient
0
1
2
3
4
5
−4
−2
0
−2
−1
0
1
2

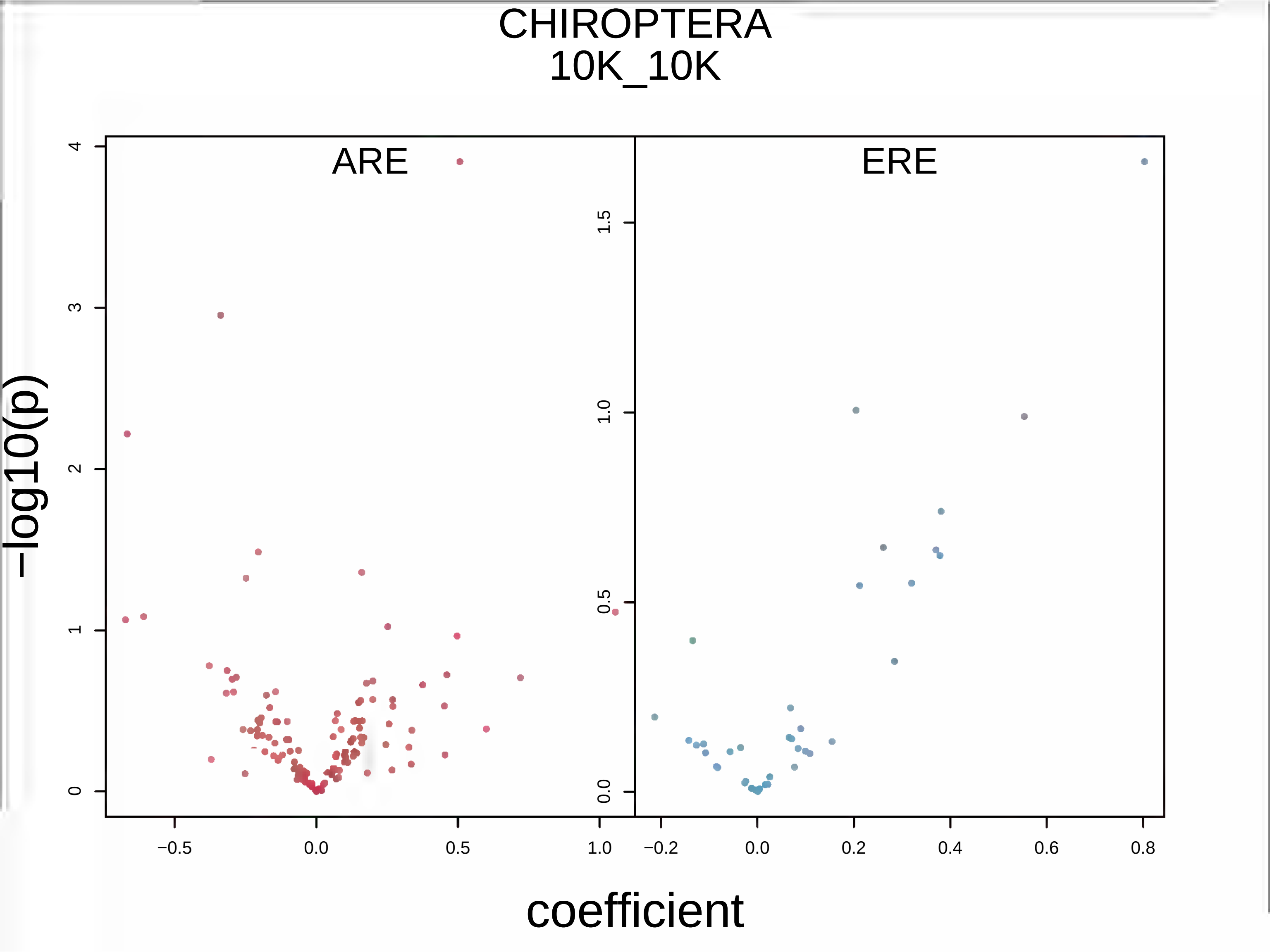
CHIROPTERA
10K_10K
ARE
ERE
−log10(p)
coefficient
0
1
2
3
4
0.0
0.5
1.0
1.5
−0.5
0.0
0.5
1.0
−0.2
0.0
0.2
0.4
0.6
0.8

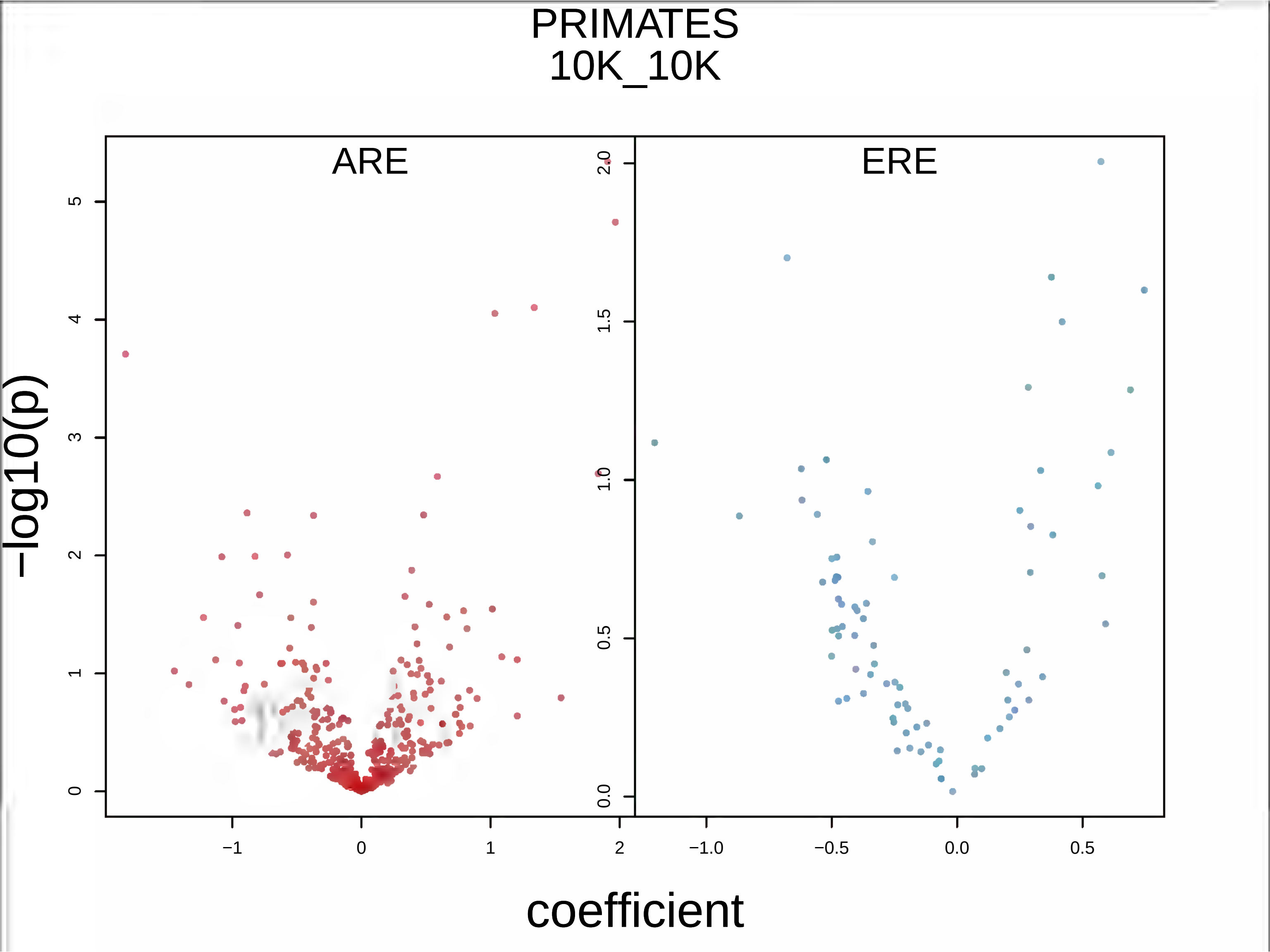

PRIMATES
10K_10K
ARE
ERE
−log10(p)
coefficient
0
1
2
3
4
5
−1
0
1
2
0.0
0.5
1.0
1.5
2.0
−1.0
−0.5
0.0
0.5

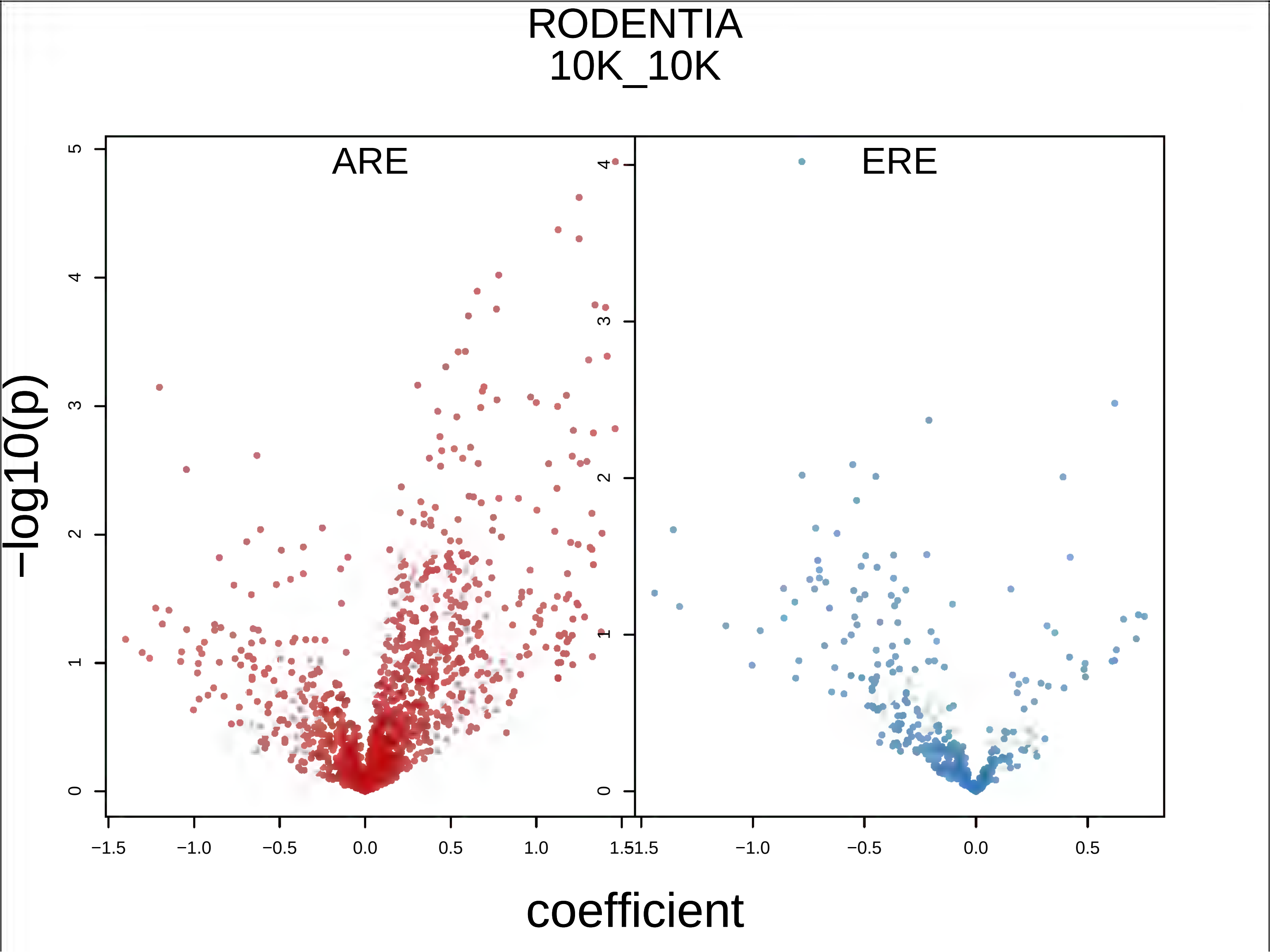

RODENTIA
10K_10K
ARE
ERE
−log10(p)
coefficient
0
1
2
3
4
5
−1.5
−1.0
−0.5
0.0
0.5
1.0
1.5

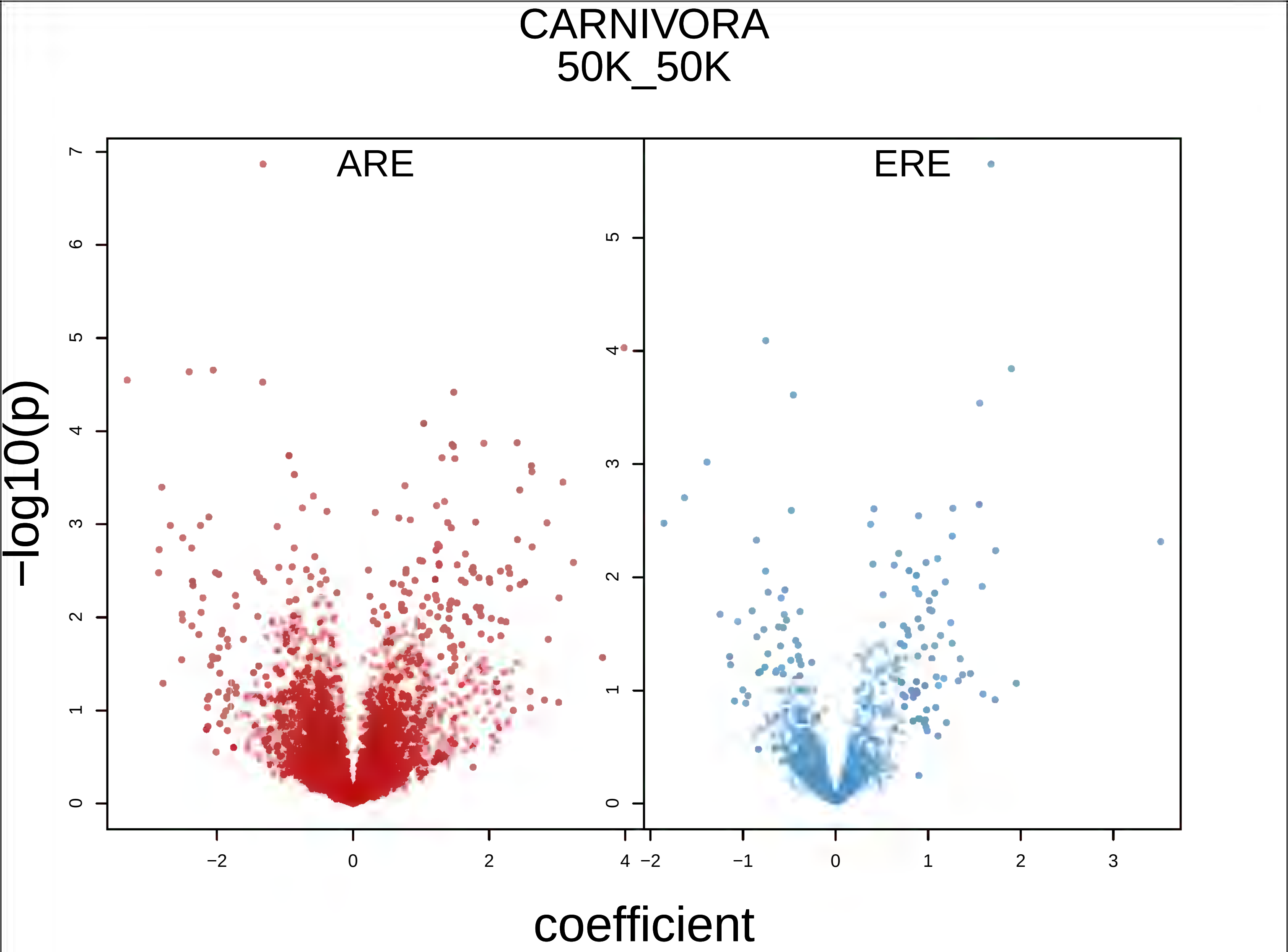
CARNIVORA
50K_50K
ARE
ERE
−log10(p)
coefficient
0
1
2
3
4
5
6
7
−2
0
2
4
−1
3

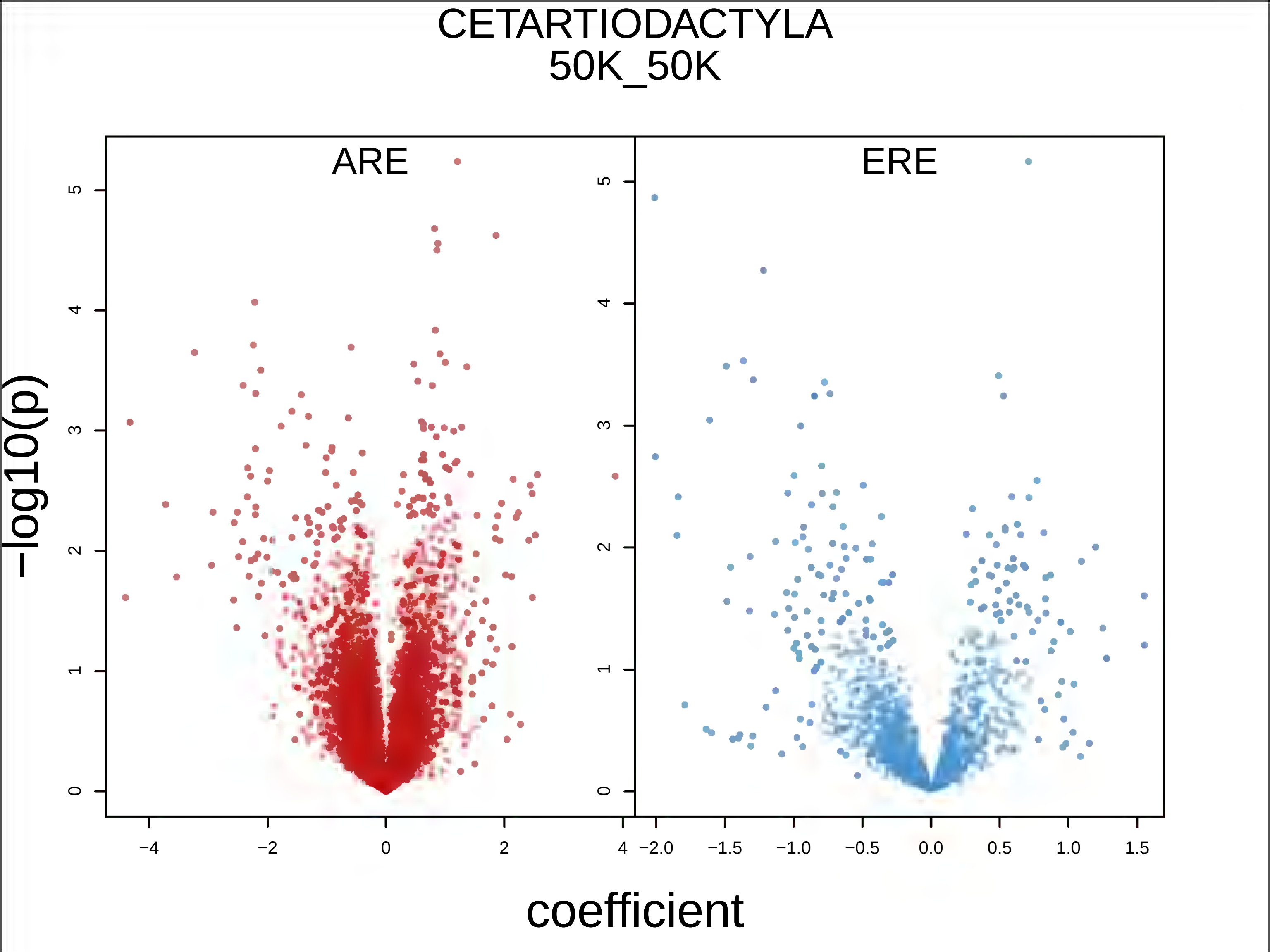

CETARTIODACTYLA
50K_50K
ARE
ERE
−log10(p)
coefficient
−4
−2
0
2
4
−2.0
−1.5
−1.0
−0.5
0.0
0.5
1.0
1.5
0
1
2
3
4
5

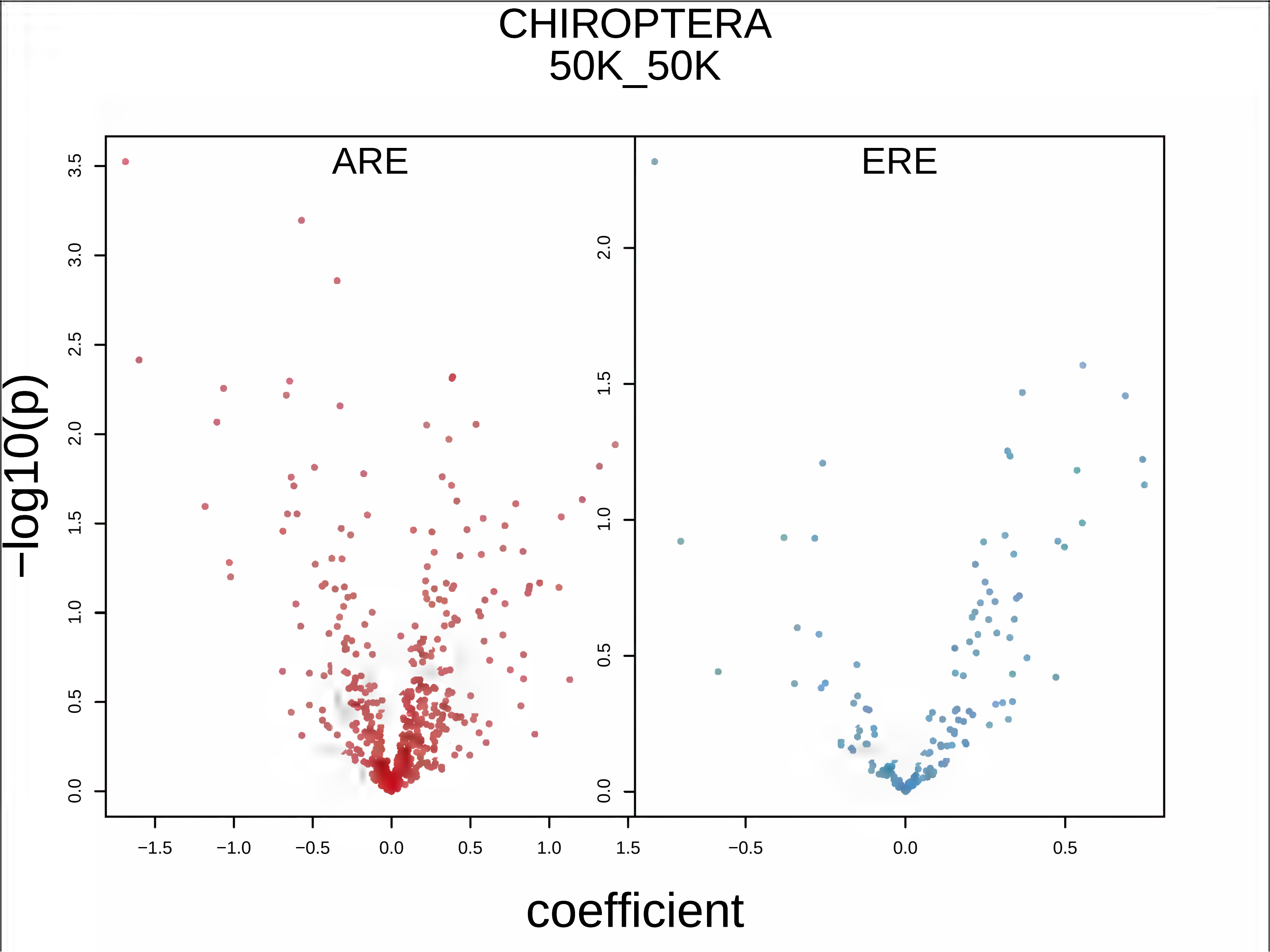

CHIROPTERA
50K_50K
ARE
ERE
−log10(p)
coefficient
0.0
0.5
1.0
1.5
2.0
2.5
3.0
3.5
−1.5
−1.0
−0.5
0.0
0.5
1.0
1.5
0.0
0.5
1.0
1.5
2.0
−0.5
0.0
0.5

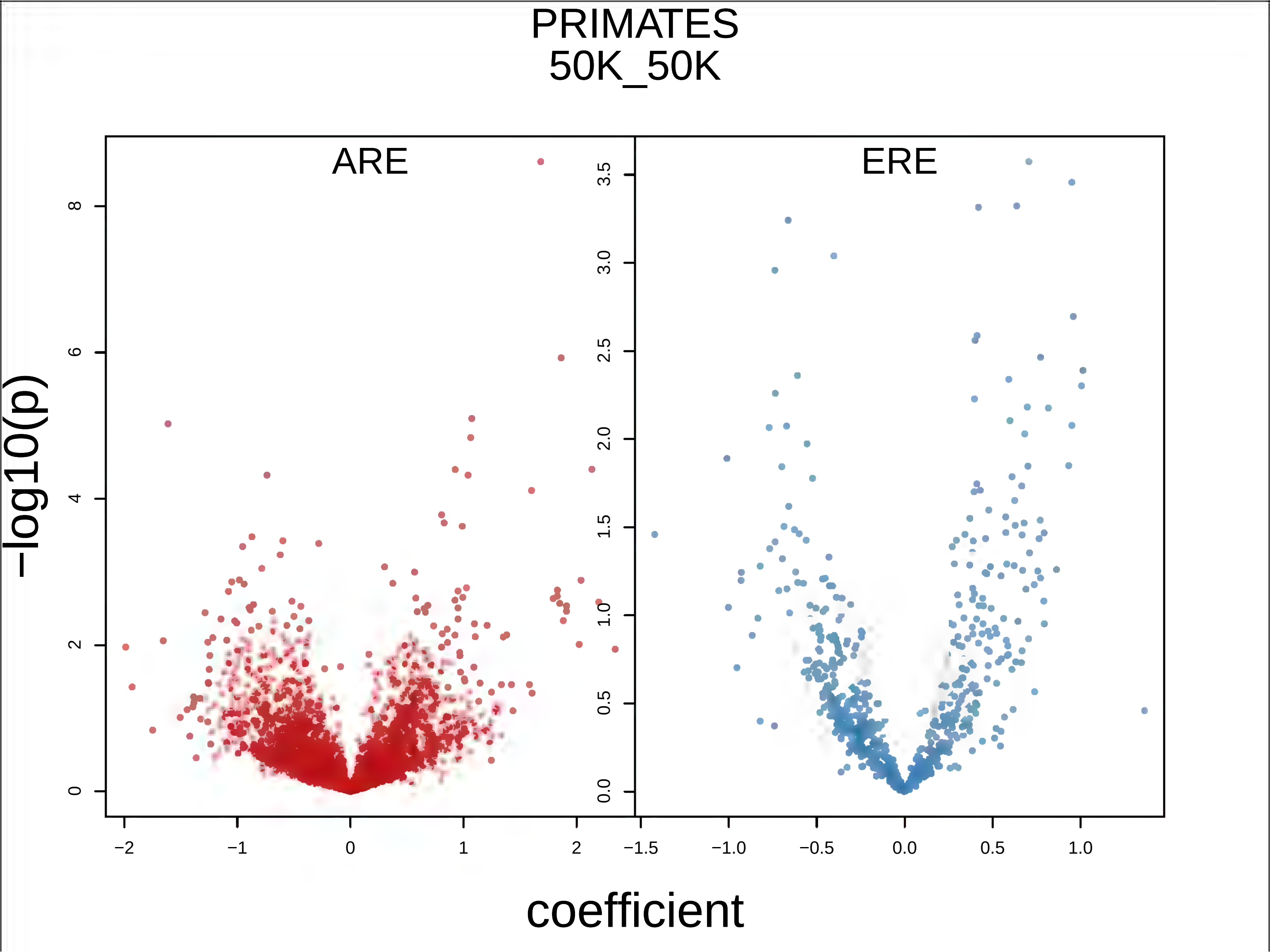

PRIMATES
50K_50K
ARE
ERE
−log10(p)
coefficient
0
2
4
6
8
0.0
0.5
1.0
1.5
2.0
2.5
3.0
3.5
−2
−1
0
1
2
−1.5
−1.0
−0.5
0.0
0.5
1.0

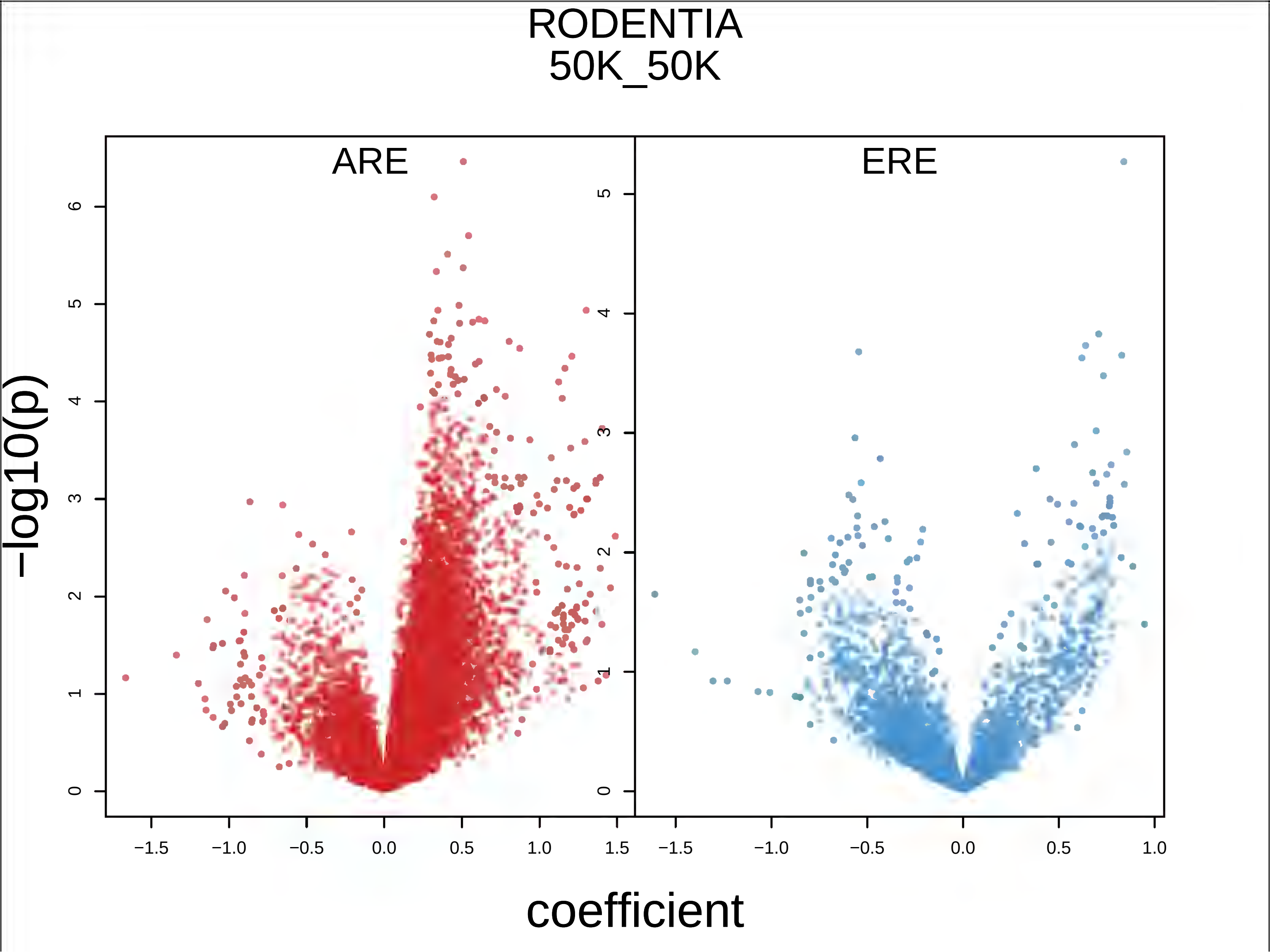
RODENTIA
50K_50K
ARE
ERE
−log10(p)
coefficient
0
1
2
3
4
5
6
−1.5
−1.0
−0.5
0.0
0.5
1.0
1.5

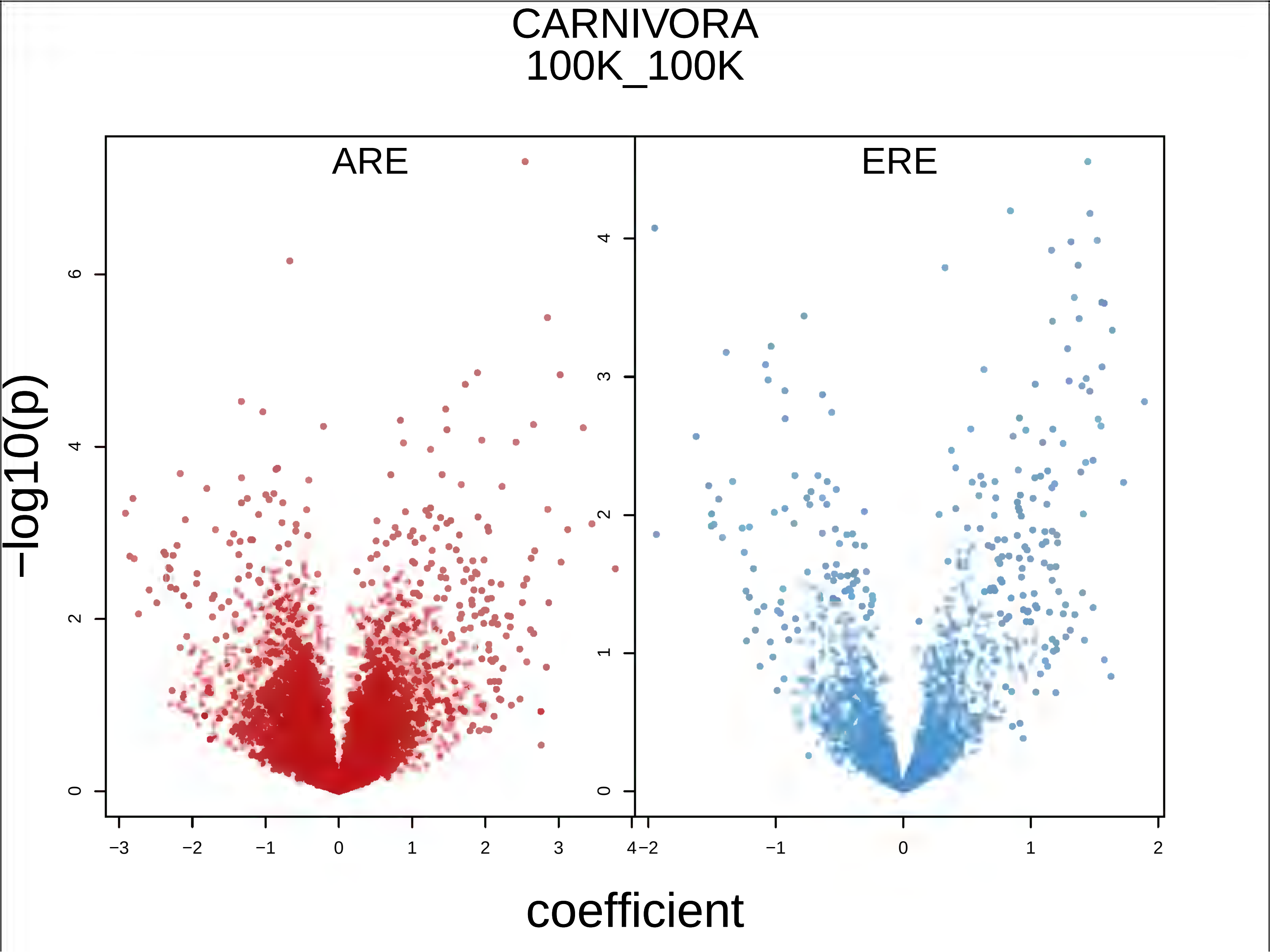
CARNIVORA
100K_100K
ARE
ERE
−log10(p)
coefficient
0
2
4
6
0
1
2
3
4
−3
−2
−1
0
1
2
3
4
−2
−1
0
1
2

CETARTIODACTYLA
100K_100K

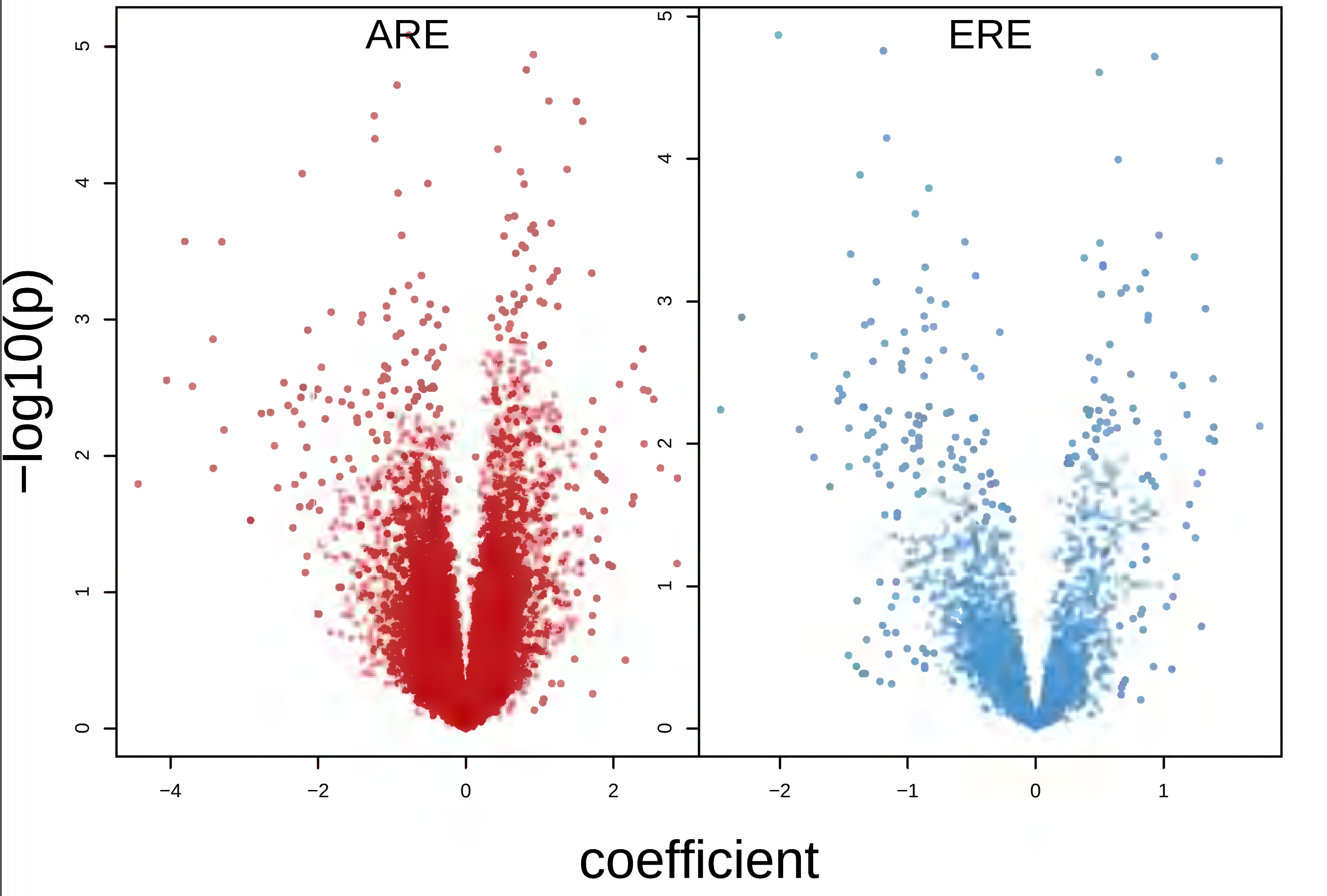

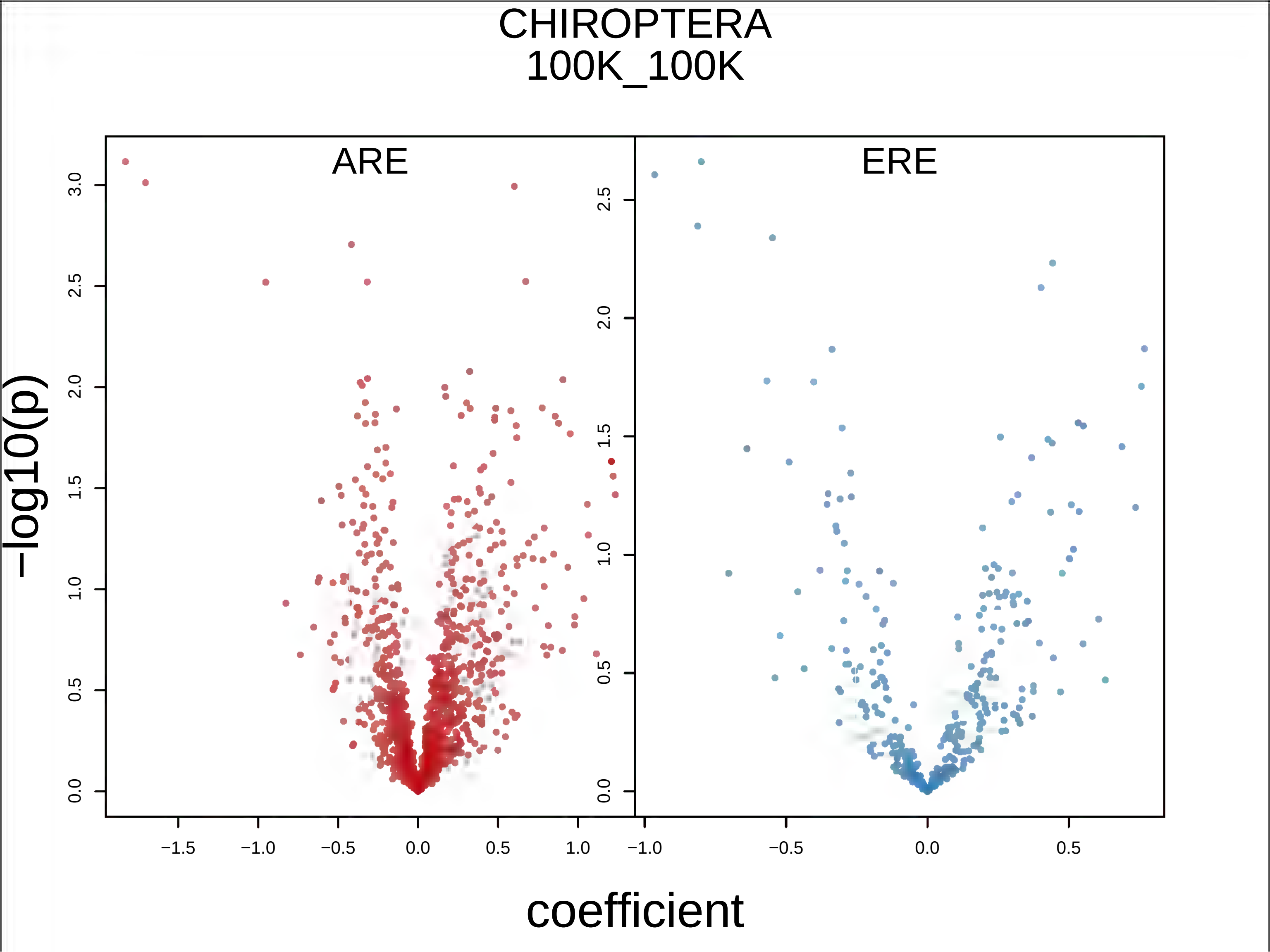

CHIROPTERA
100K_100K
ARE
ERE
−log10(p)
coefficient
3.0
2.5
2.0
1.5
1.0
0.5
0.0
−1.5
−1.0
−0.5
0.0
0.5
1.0

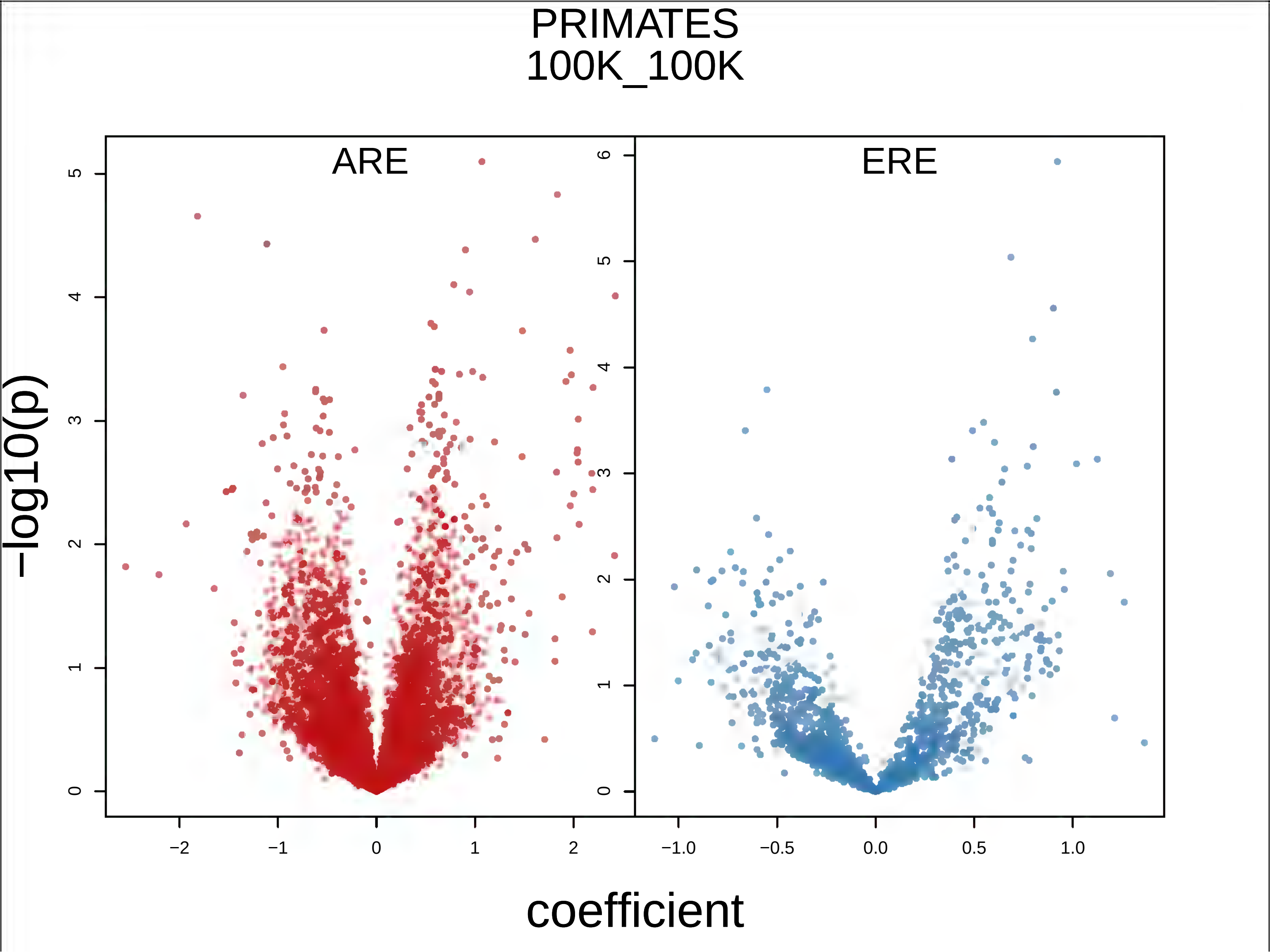
PRIMATES
100K_100K
ARE
ERE
−log10(p)
coefficient
0
1
2
3
4
5
6
−2
−1
0
1
2
−1.0
−0.5
0.0
0.5
1.0

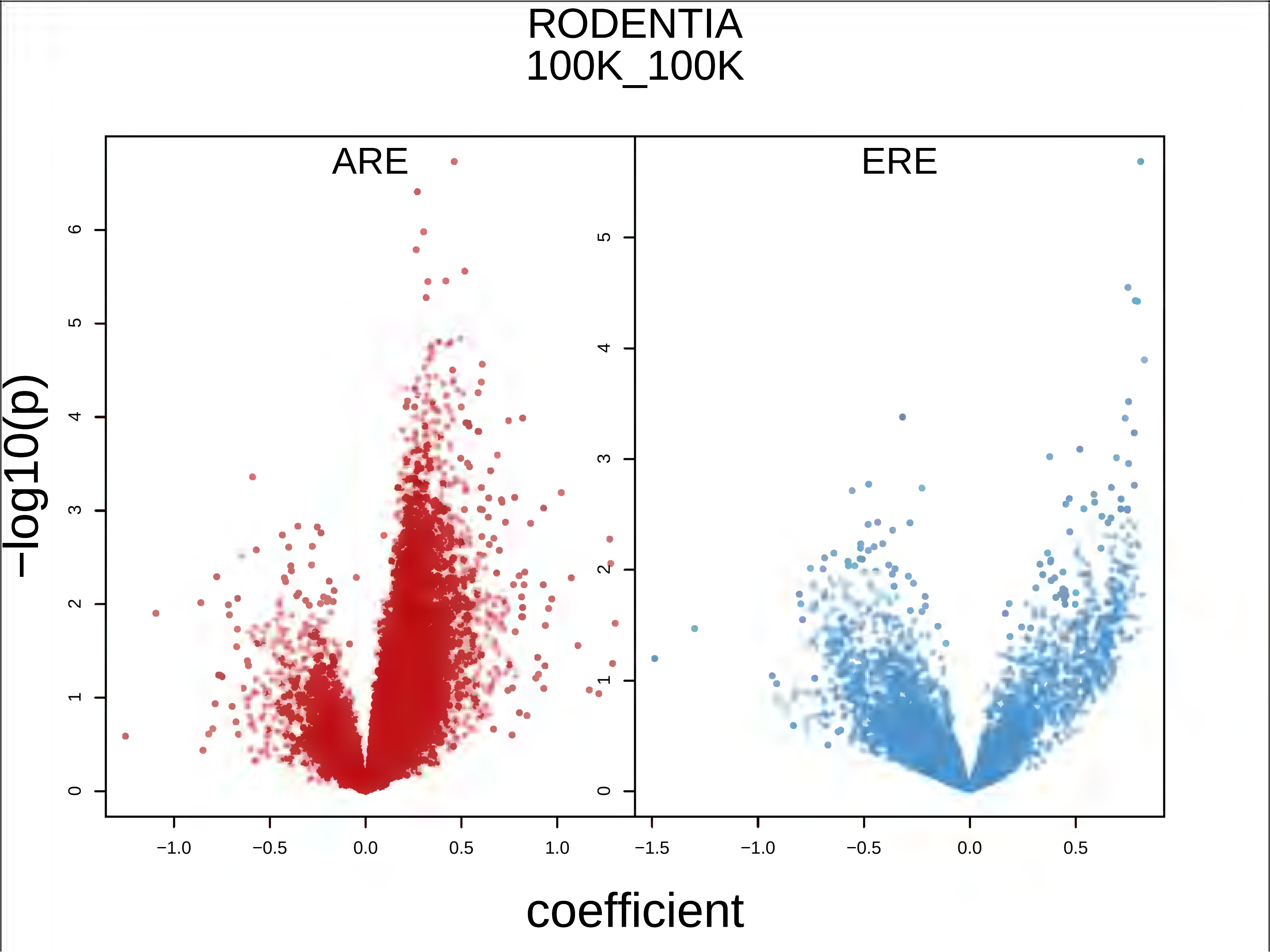
RODENTIA
100K_100K
ARE
ERE
−log10(p)
coefficient
0
1
2
3
4
5
6
−1.0
−0.5
0.0
0.5
1.0
−1.5

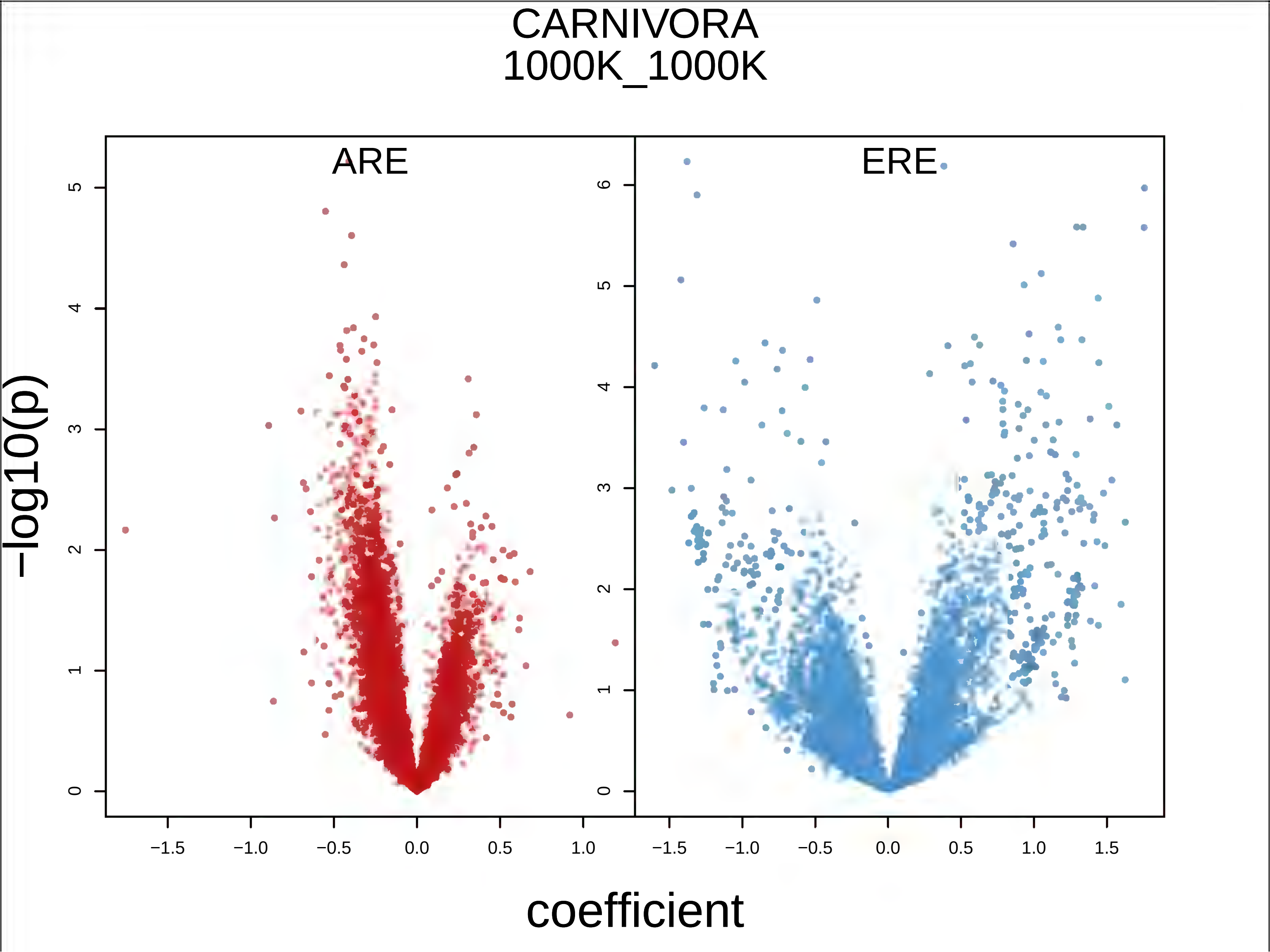

CARNIVORA
1000K_1000K
ARE
ERE
−log10(p)
coefficient
0
1
2
3
4
5
6
−1.5
−1.0
−0.5
0.0
0.5
1.0
1.5

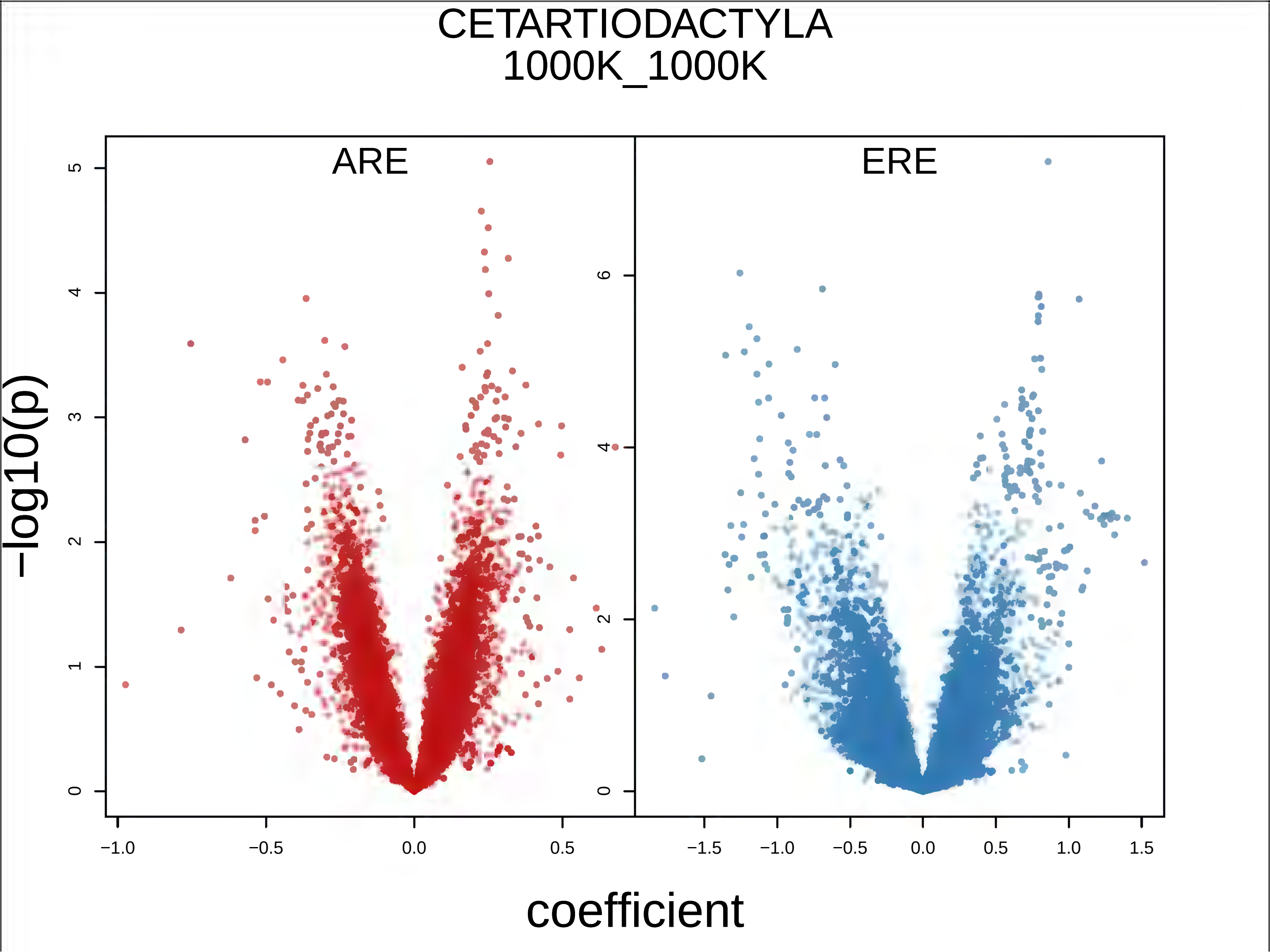

CETARTIODACTYLA
1000K_1000K
ARE
ERE
−log10(p)
coefficient
0
1
2
3
4
5
6
−1.0
−0.5
0.0
0.5
1.0
1.5
−1.5

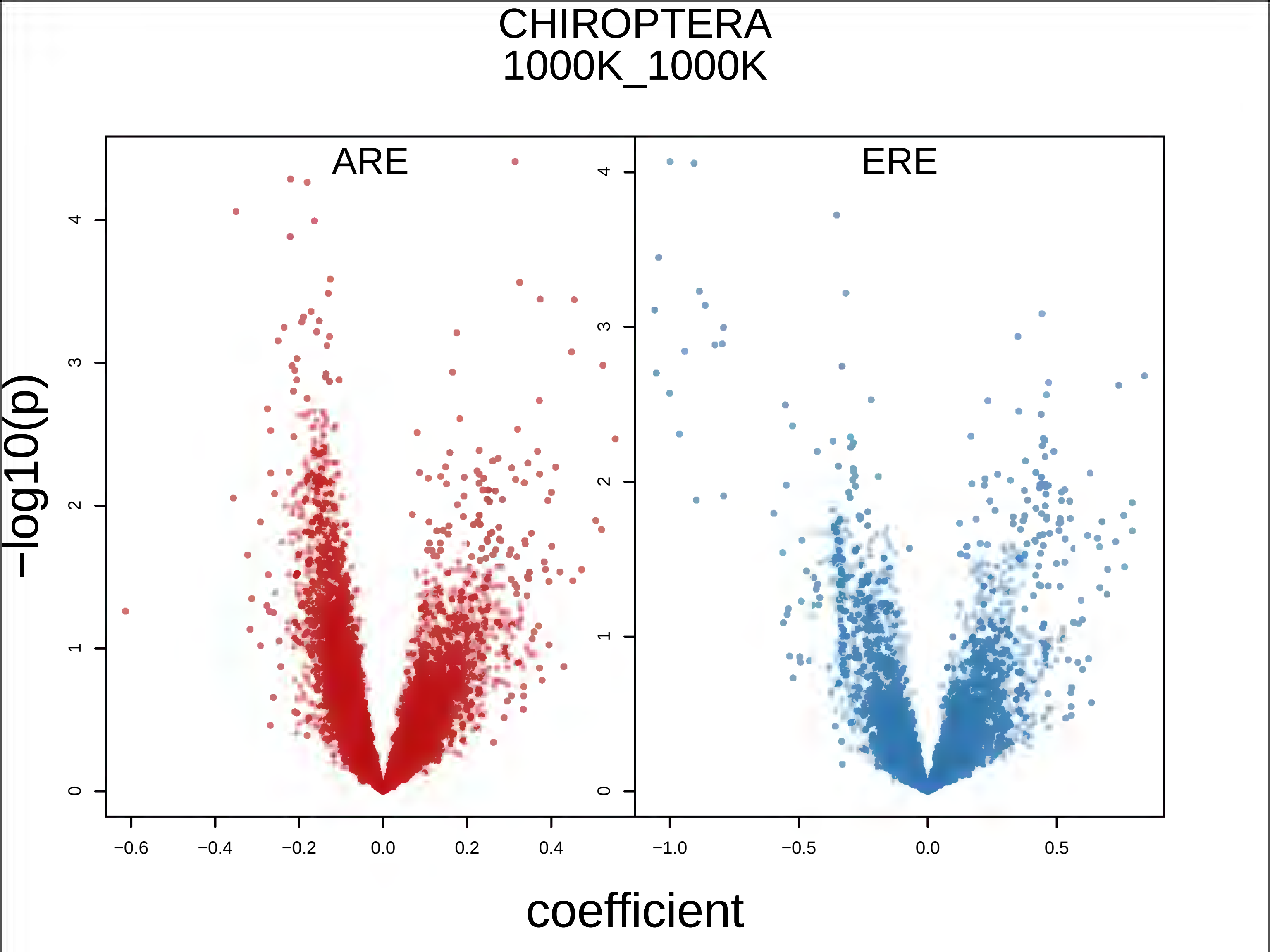
CHIROPTERA
1000K_1000K
ARE
ERE
−log10(p)
coefficient
0
1
2
3
4
−0.6
−0.4
−0.2
0.0
0.2
0.4
−1.0
−0.5
0.0
0.5

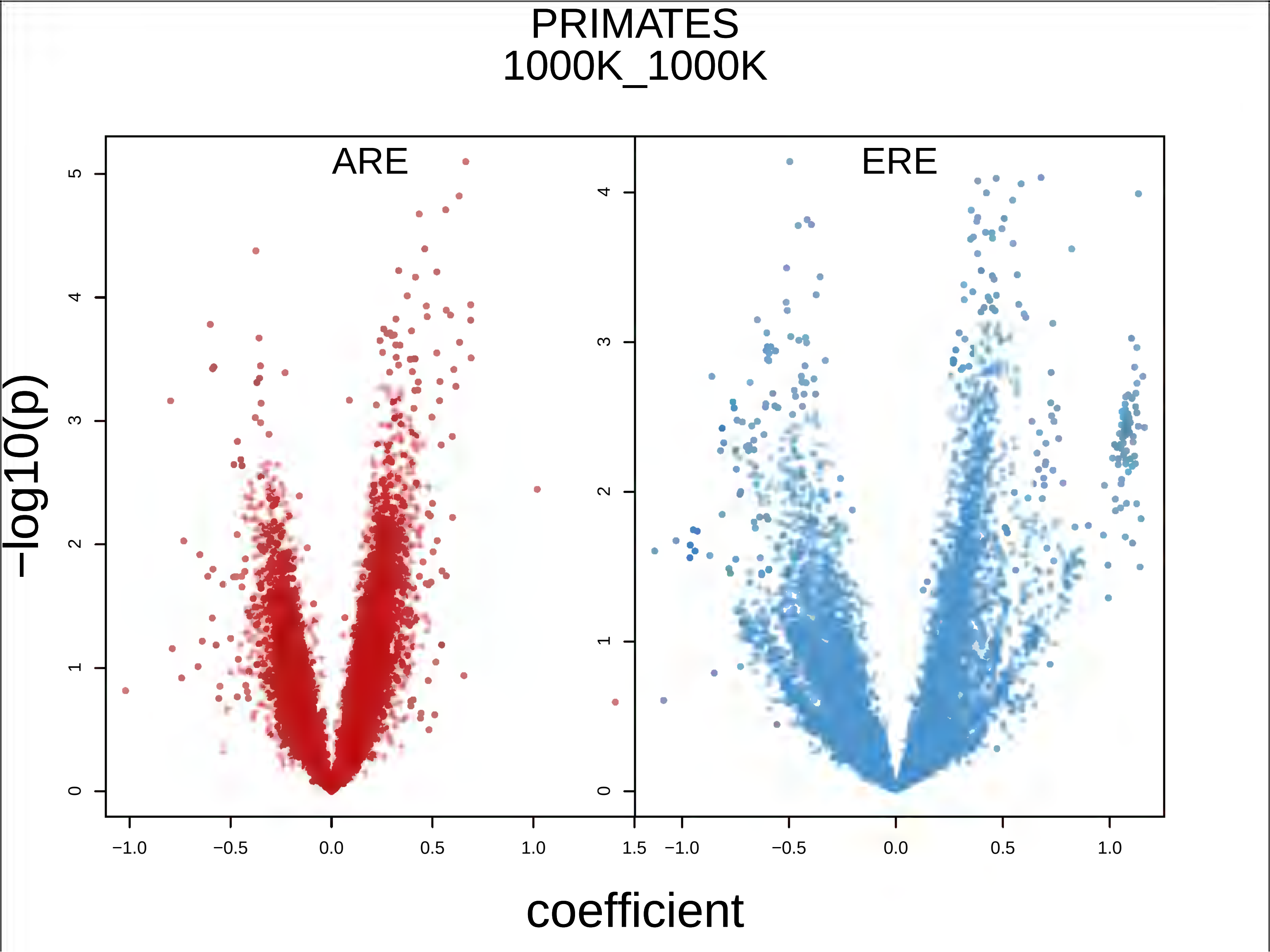
PRIMATES
1000K_1000K
ARE
ERE
−log10(p)
coefficient
0
1
2
3
4
5
−1.0
−0.5
0.0
0.5
1.0
1.5

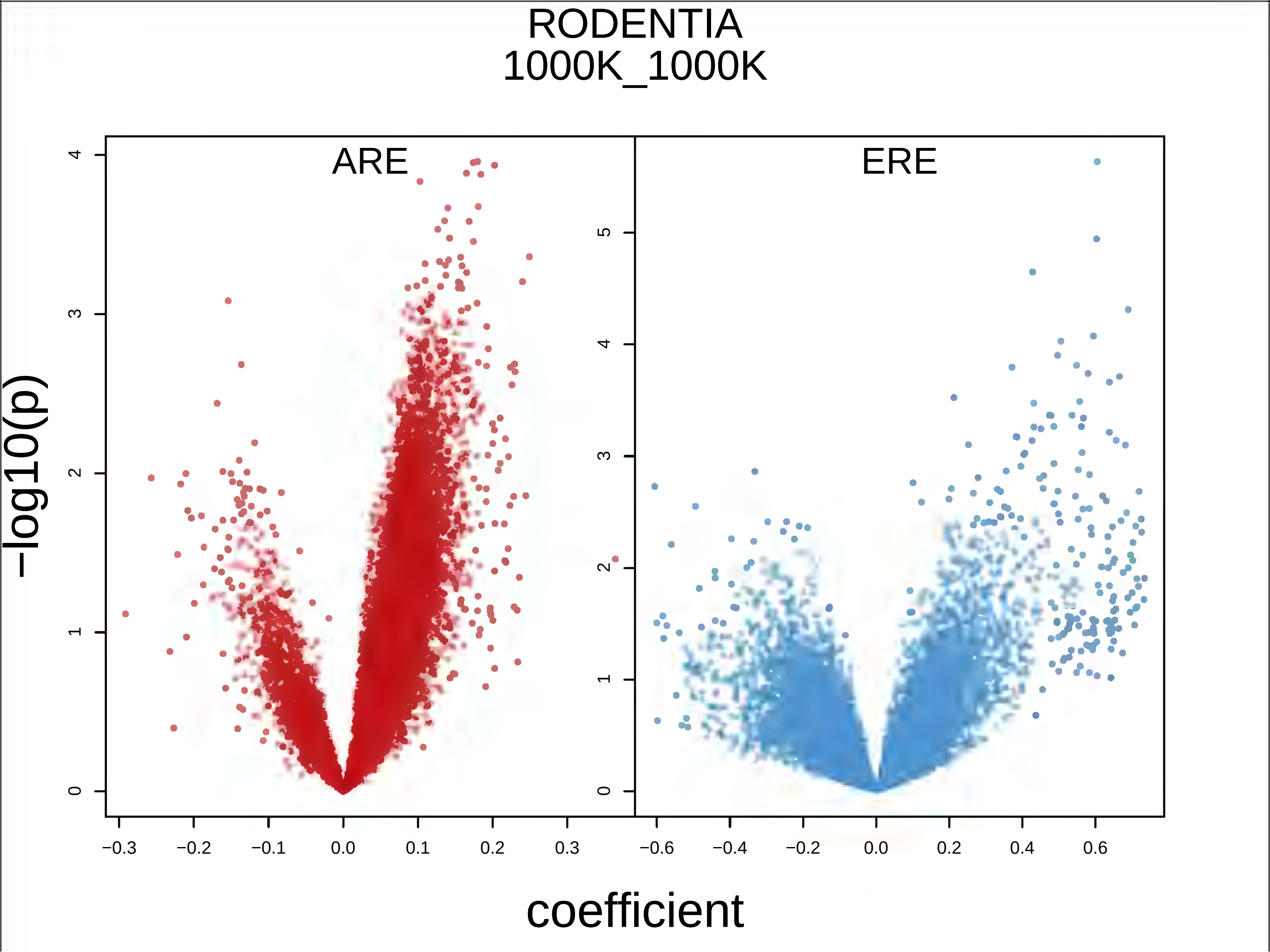
RODENTIA
1000K_1000K
ARE
ERE
−log10(p)
coefficient
0
1
2
3
4
5
−0.3
−0.2
−0.1
0.0
0.1
0.2
0.3
−0.6
−0.4
−0.2
0.0
0.2
0.4
0.6
